# The osteogenetic activities of mesenchymal stem cells in response to Mg^2+^ ions and inflammatory cytokines: a numerical approach using fuzzy logic controllers

**DOI:** 10.1371/journal.pcbi.1010482

**Published:** 2022-09-15

**Authors:** Jalil Nourisa, Berit Zeller-Plumhoff, Regine Willumeit-Römer

**Affiliations:** Helmholtz Zentrum Hereon, Institute of Metallic Biomaterials, Geesthacht, Germany; Queensland University of Technology, AUSTRALIA

## Abstract

Magnesium (Mg^2+^) ions are frequently reported to regulate osteogenic activities of mesenchymal stem cells (MSCs). In this study, we propose a numerical model to study the regulatory importance of Mg^2+^ ions on MSCs osteoblastic differentiation in the presence of an inflammatory response. A fuzzy logic controller was formulated to receive the concentrations of Mg^2+^ ions and the inflammatory cytokines of TNF-α, IL-10, IL-1β, and IL-8 as cellular inputs and predict the cells’ early and late differentiation rates. Five sets of empirical data obtained from published cell culture experiments were used to calibrate the model. The model successfully reproduced the empirical data regarding the concentration- and phase-dependent effect of Mg^2+^ ions on the differentiation process. In agreement with the experiments, the model showed the stimulatory role of Mg^2+^ ions on the early differentiation phase, once administered at low concentration, and their inhibitory role on the late differentiation phase. The numerical approach used in this study suggested 6–8 mM as the most effective concentration of Mg^2+^ ions in promoting the early differentiation process. Also, the proposed model sheds light on the fundamental differences in the behavioral properties of cells cultured in different experiments, e.g. differentiation rate and the sensitivity of the cultured cells to stimulatory signals such as Mg^2+^ ions. Thus, it can be used to interpret and compare different empirical findings. Moreover, the model successfully reproduced the nonlinearities in the concentration-dependent role of the inflammatory cytokines in early and late differentiation rates. Overall, the proposed model can be employed in studying the osteogenic properties of Mg-based implants in the presence of an inflammatory response.

## Author summary

Magnesium (Mg) is an attractive material for bone implants as it fully degrades after implantation, saving pain and cost of the second surgery for implant removal. To advance its application in the orthopedic industry, it is paramount to fully understand the biological impact of the degradation products, in particular Mg^2+^ ions. Here, we propose a computer model to study the effects of Mg^2+^ ions on bone regeneration. The model focuses on stem cells and includes both the direct stimulation effects of Mg^2+^ ions on cells and the indirect stimulus through the inflammatory system. The proposed model successfully reproduced the experimental data of five different studies. The model additionally highlighted differences amongst different experiments in terms of the cellular response to Mg^2+^ ions. The proposed system therefore provides an important addition to the field of Mg implant research.

## 1 Introduction

**Fig 1:**
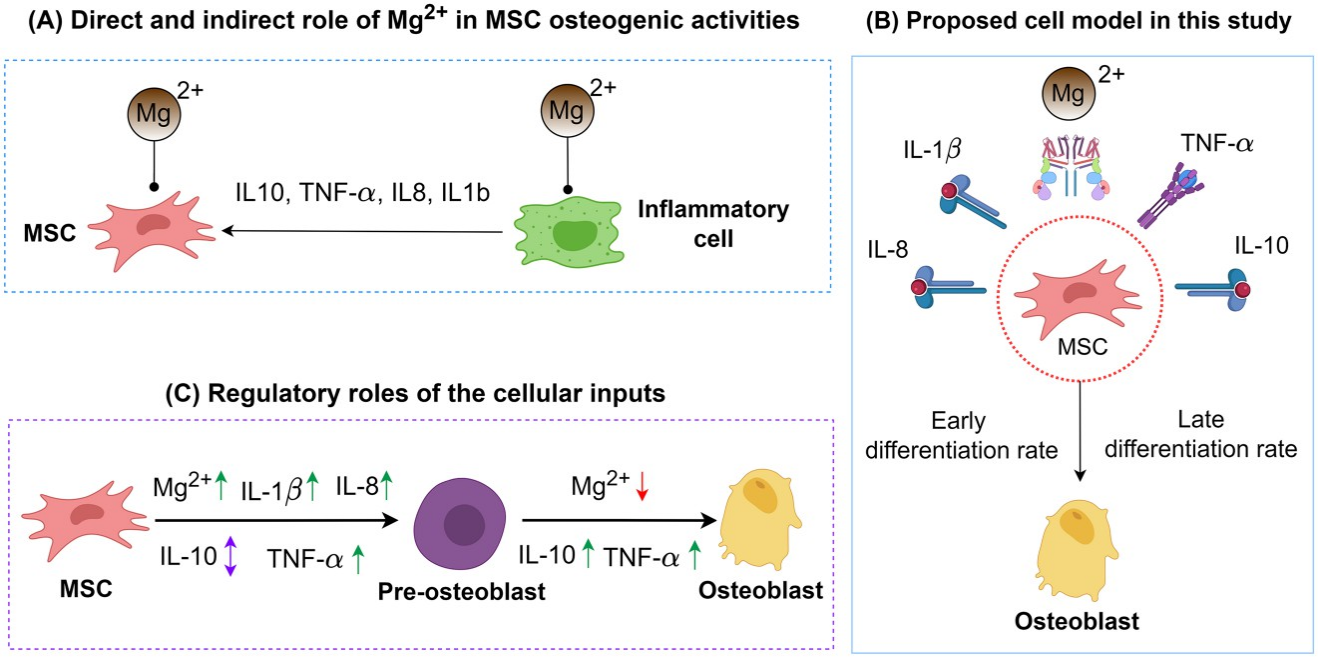
(A) Mg^2+^ ions regulate MSC differentiation in both direct and indirect ways. (B) the cell model proposed in this study receives five cellular signals and predicts early and late differentiation rates. (C) the regulatory role of Mg^2+^ ions and the inflammatory cytokines on the early and late osteogenic differentiation. Upwards arrays in green indicate stimulatory roles; downwards arrays in red indicate inhibitory roles; up-down arrows in purple indicate dose-dependent effect. BioRender.com is used to create some elements of the graph.

Magnesium (Mg)-based biomaterials are an attractive choice in the orthopedic industry due to their biodegradability and superior osteogenic capacity compared to non-degradable metallic implants e.g., titanium alloys [1–3]. Mg^2+^ ions released in the implantation site due to the degradation process modulate a wide range of physiological processes involved in bone fracture healing, in particular osteogenesis [1], [4]. The mediatory effects of Mg^2+^ ions on osteogenic differentiation can be evaluated in two aspects (see Fig 1A). First, Mg^2+^ ions directly modulate multiple signaling pathways associated with the differentiation of mesenchymal stem cells (MSCs) to osteoblasts [5], [6]; Mg^2+^ ions are shown to activate MAPK/ERK and Wnt/ β-catenin signaling pathways which are associated with osteogenic differentiation [7]. Secondly, Mg^2+^ ions mediate the inflammatory response and thereby indirectly guide osteogenesis [8–10].

The inflammatory response is known to play a pivotal role in all stages of bone tissue regeneration [11]–[13]. In particular, macrophages are repeatedly shown to guide osteogenesis [12], [14], [11]. During the healing process, macrophages obtain different functional roles ranging from pro-inflammatory M1 to anti-inflammatory M2 and secrete a multitude of cytokines essential for the osteogenic ability of MSCs [14], [15]. Empirical data have shown the significance of Mg^2+^ ions in modulating macrophage polarization and cytokines production [9], [10], [16], [17]. For instance, Qiao et al. [17] applied Mg^2+^ ions to macrophages in a cell culture environment and observed an upregulation in the production of interleukin (IL)-8, which is an important factor in the osteogenic differentiation of MSCs. Such an indirect influence of Mg^2+^ ions on osteogenesis is reported to even overweight the direct regulatory effect of Mg^2+^ ions on MSCs [17].

Although Mg^2+^ ions are generally shown to promote osteogenesis, multiple studies have reported their detrimental effects on tissue regeneration [8], [18]. Such a conflicting finding implies an incomplete understanding of the role of Mg^2+^ ions in the complex process of fracture healing. In order to effectively design a Mg-based implant, it is paramount to fully understand the bioregulatory role of Mg^2+^ ions on tissue regeneration in the presence of other signaling factors, in particular inflammatory reactions [4], [19][20]. So far, the experimental approach has been the primary method in investigating the physiological roles of Mg^2+^ ions. In recent years, numerous studies have shown the importance of numerical investigation in the study of biological systems and bone-implant design [21–25]. Fuzzy logic (FL) has been given a special attention in quantitative modelling of biological systems with uncertain kinetic data [26][27]. Aldridge et al [28] used FL in describing the dynamics of intracellular pathways of human colon carcinoma cells associated with different growth factors and insulin receptors. Their FL-based simulations successfully produced several predictions of pathways crosstalk and regulation [28]. They also proposed a relationship between MK2 and ERK pathways which was unknown previously [28]. Wang et al [29] and Niemeyer et al [30] employed FL to simulate the transitional formation of different tissues during bone regeneration. They have successfully shown the patterns of bone tissue regeneration under various mechanical environment [29], [30].

Recently, we proposed a fuzzy agent-based model to numerically study the proliferation and osteogenic differentiation of MSCs in response to Mg^2+^ ions [31]. We showed that Mg^2+^ ions within 3-6mM concentration have the highest stimulation effect on cell population growth. The model also captured the stimulatory role of Mg^2+^ ions on early differentiation and its inhibitory effect on the late differentiation process. In this study, as an extension of our previous model, we propose a FL-based model to investigate the osteogenic response of MSCs to Mg^2+^ ions in tandem with the inflammatory cytokines of tumor necrosis factor alpha (TNF-α), interleukin 10 (IL-10), interleukin 1 beta (IL-1β), and IL-8. In the present model, the previously proposed controller for the Mg^2+^ ions is further extended to include the inhibitory role of Mg^2+^ ions on the early osteogenic response once the concentrations is below the physiological level. In addition, five more controllers are presented to account for the regulatory roles of the given inflammatory cytokines.

## 2 Results

### 2.1 The overview of the model formulation

In this study, the proposed cell model receives five cellular inputs and predicts the osteogenic differentiation rates (see Fig 1B). The choice of cytokines is based on their importance in osteogenesis and their involvement in the interplay of Mg^2+^ ions with the inflammatory cells, as elaborated in section 4.1. Since the cellular inputs can regulate the early and late differentiation process differently (Fig 1C), two distinguished processes of early- and late differentiation rates are calculated by the model. A FL controller is designed to act as the core calculator of the cell model. In order to define fuzzy rules, we gathered the available information in the literature regarding the regulatory role of the cellular inputs (see section 4.1). A complete introduction to the development of the FL controller is given in section 5. The formulation of the model generated 30 unknown parameters given in S3 Table. We estimated the values of these parameters using a calibration process by employing differential evolution (DE) [32]. The published data of five cell-culture experiments were used for this purpose (see section 5.5). These experiments reported the bioregulatory effect of Mg2+ ions and different inflammatory cytokines on the osteoblastic differentiation of MSC. In these reports, alkaline phosphate (ALP) was used as the early differentiation marker, while osteocalcin (OC) and alkaline red staining (ARS) were the late differentiation markers. The parameter estimation process was carried out to maximize the fitness value, which is defined as the normalized absolute difference between the simulation results (S) and the empirical observations (E) as |*R*^2^ = |E-S|/E. The calibration process was conducted on the dataset of each experiment individually, encoded as C1 to C5, as well as on the combined data of all experiments, encoded as C1-5. Once the model was calibrated, we investigated the sensitivity of the model to the parameters using large-scale simultaneous perturbations (LSSP) and small-scale individual perturbations (SSIP). The details of the calibration process and the sensitivity analysis are given in section 5.6.

### 2.2 Simulation results versus empirical observations

#### 2.2.1 Study 1

**Fig 2:**
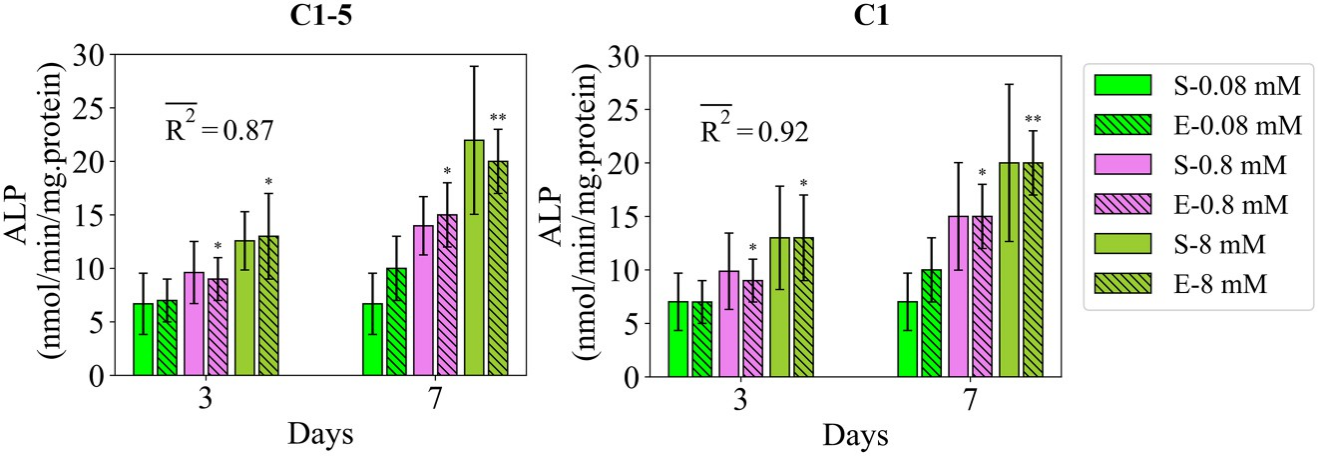
Fits of the model calibrated by C1-5 and C1 to the empirical data of study 1. Bars indicate the simulations (S-) and the corresponding empirical data (E-) for increasing Mg^2+^ ion concentrations. The error bars on the empirical data show the standard deviations. The error bars on the simulation results show the standard deviations obtained during SSIP, i.e. 15% alteration in the estimated parameter values. Stars indicate the statistically significant differences between values given for the empirical data compared to the control, i.e. Mg^2+^ ion concentration of 0.08 mM (p < 0.05 = *; p < 0.01 = **). $\overset{-}{R^{2}}$ is the average fitness value of the simulations for the given measurement item.

Study 1 reports the regulatory effect of Mg^2+^ ions on the early differentiation rate, measured by ALP [17]. The fits of the simulation results to the data of study 1 are presented in Fig 2. The empirical data suggest that the application of Mg^2+^ ions at the concentration of 8 mM increases the early differentiation rate at both measurement days of 3 and 7 compared to the physiological concentration of 0.8 mM (see Fig 2). Contrarily, the application of Mg^2+^ ions at the concentration of 0.08 mM decreases the early differentiation rate (see Fig 2). These observations were correctly captured by the model calibrated by C1-5 with a fitness value of 0.84. Once calibrated by C1, the model was able to closely reproduce the reported measurements with the average fitness value of 0.92. The variations in the simulation results of study 1 due to SSIP are given in Fig 2 for each measurement item.

#### 2.2.2 Study 2

**Fig 3:**
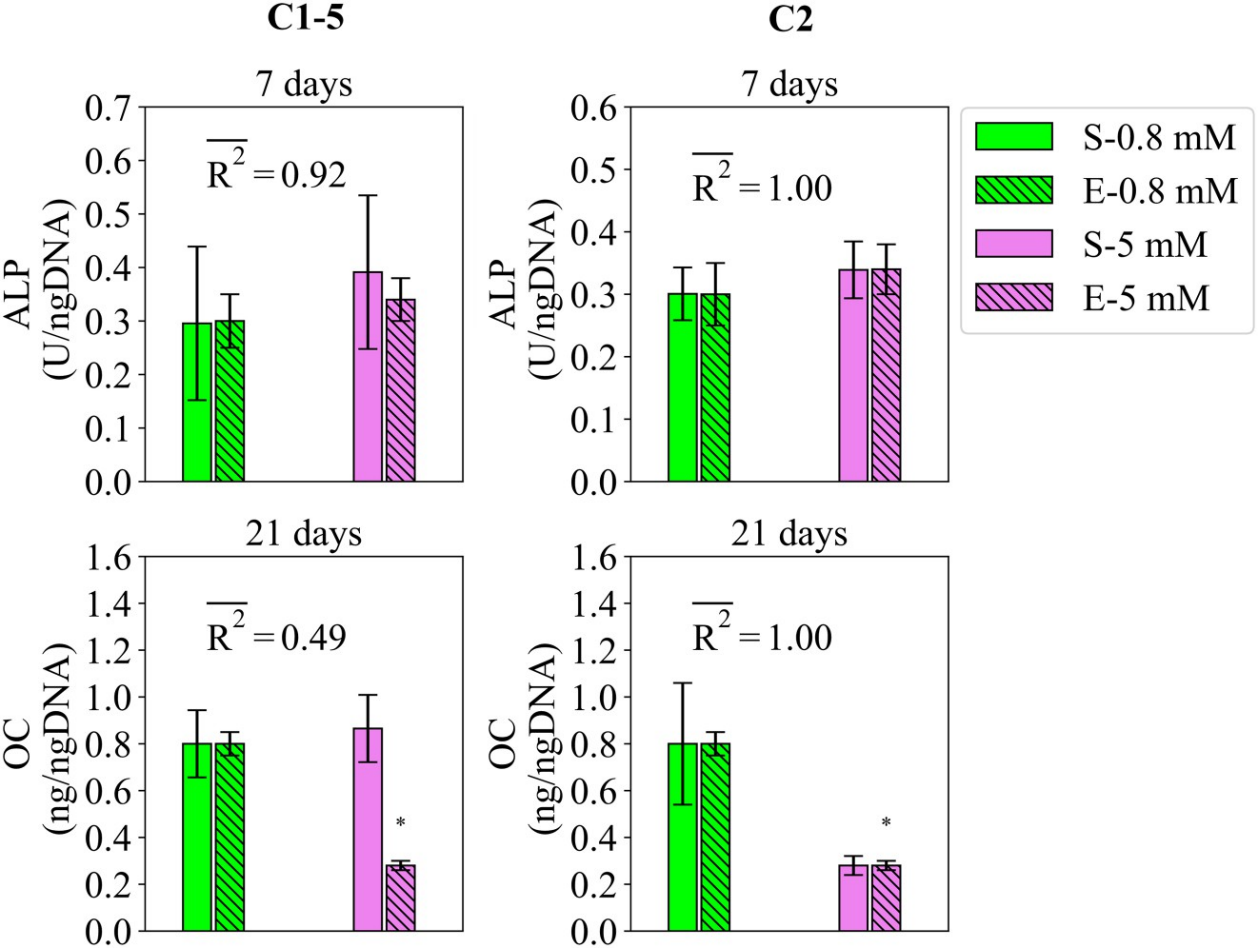
Fits of the model calibrated by C1-5 and C2 to the empirical data of study 2. Bars indicate the simulations (S-) and the corresponding empirical data (E-) for increasing Mg^2+^ ion concentrations. The quantities of ALP and OC are reported at day 7 and 21, repectively. The error bars on the empirical data shows the standard deviations. The error bars on the simulation results show the standard deviations obtained during SSIP, i.e. 15% alteration in the estimated parameter values. Stars indicate the statistically significant differences between values given for the empirical data compared to the control, i.e. Mg^2+^ ion concentration of 0.8 mM (p < 0.05 = *). $\overset{-}{R^{2}}$ is the average fitness value of the simulations for the given measurement item.

Study 2 reports the regulatory effects of Mg^2+^ ions on the early and late differentiation rates, measured by ALP and OC, respectively [6]. The fits of the model to the data of study 2 are given in Fig 3. The data suggests that by increasing Mg^2+^ ions from 0.8 to 5 mM, the early differentiation rate increases while the late differentiation rate decreases. The model calibrated by C1-5 was able to capture the former but not the latter. The mean fitness values obtained during Effects of magnesium degradation products on mesenchymal stem cell fate and osteoblastogenesis C1-5 were 0.94 for ALP and 0.49 for OC (see Fig 3). Once calibrated by C2, the model was capable of correctly reproducing both trends and exact values with the fitness values of 1 for both markers (see Fig 3). The variations in the simulation results of study 2 due to SSIP are given in Fig 3 for different measurement items.

#### 2.2.3 Study 3

**Fig 4:**
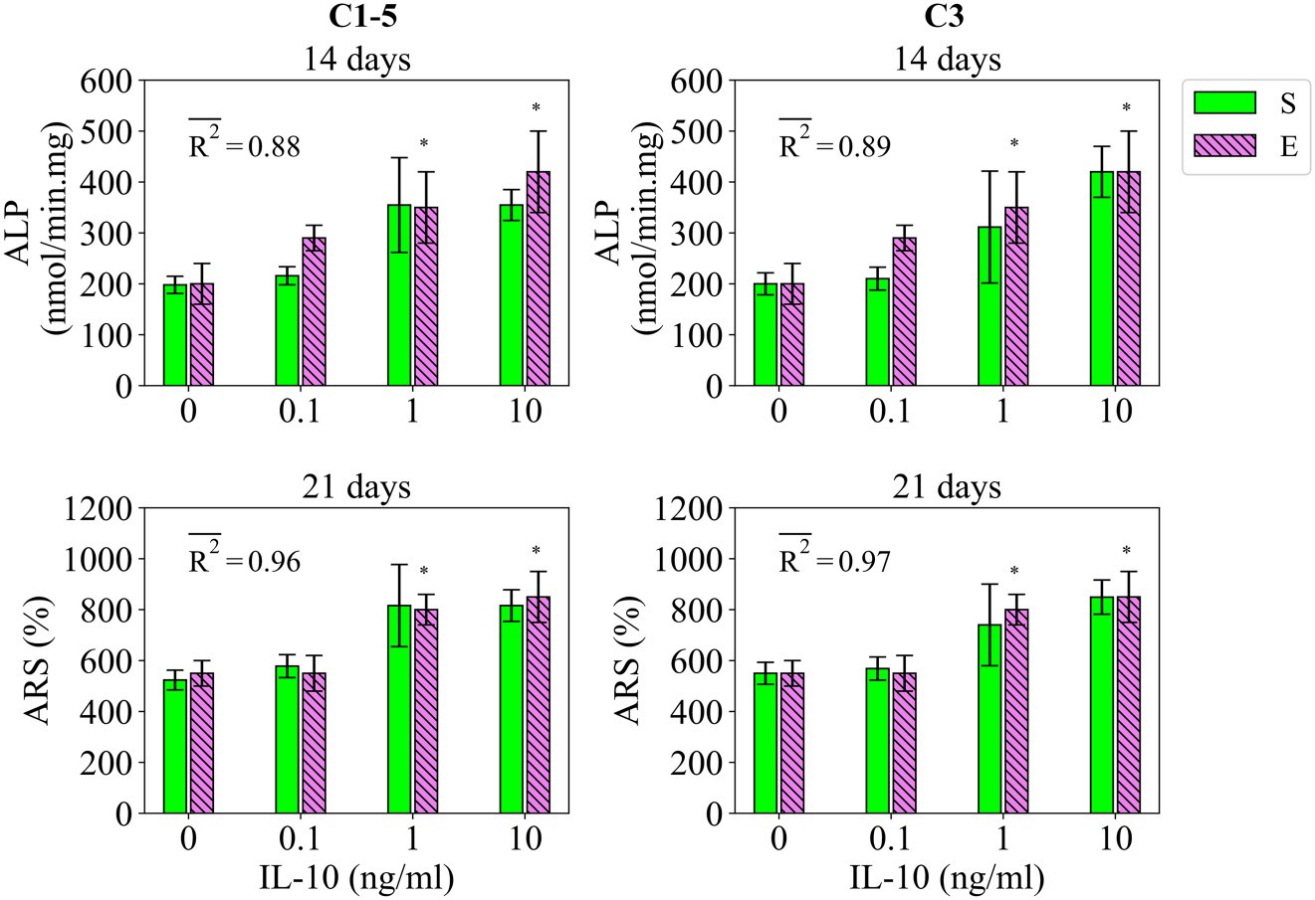
Fits of the model calibrated by C1-5 and C3 to the empirical data of study 3 for the case of IL-10. Bars indicate the simulations (S) and the corresponding empirical data (E). The quantities of ALP and ARS are reported at day 14 and 21, respectively. The error bars on the empirical data shows the standard deviations. The error bars on the simulation results show the standard deviations obtained during SSIP, i.e. 15% alteration in the estimated parameter values. Stars indicate the statistically significant differences between values given for the empirical data compared to the control, i.e. the applied concentration of 0 ng/ml (p < 0.05 = *). $\overset{-}{R^{2}}$ is the average fitness value of the simulations for the given measurement item.

**Fig 5:**
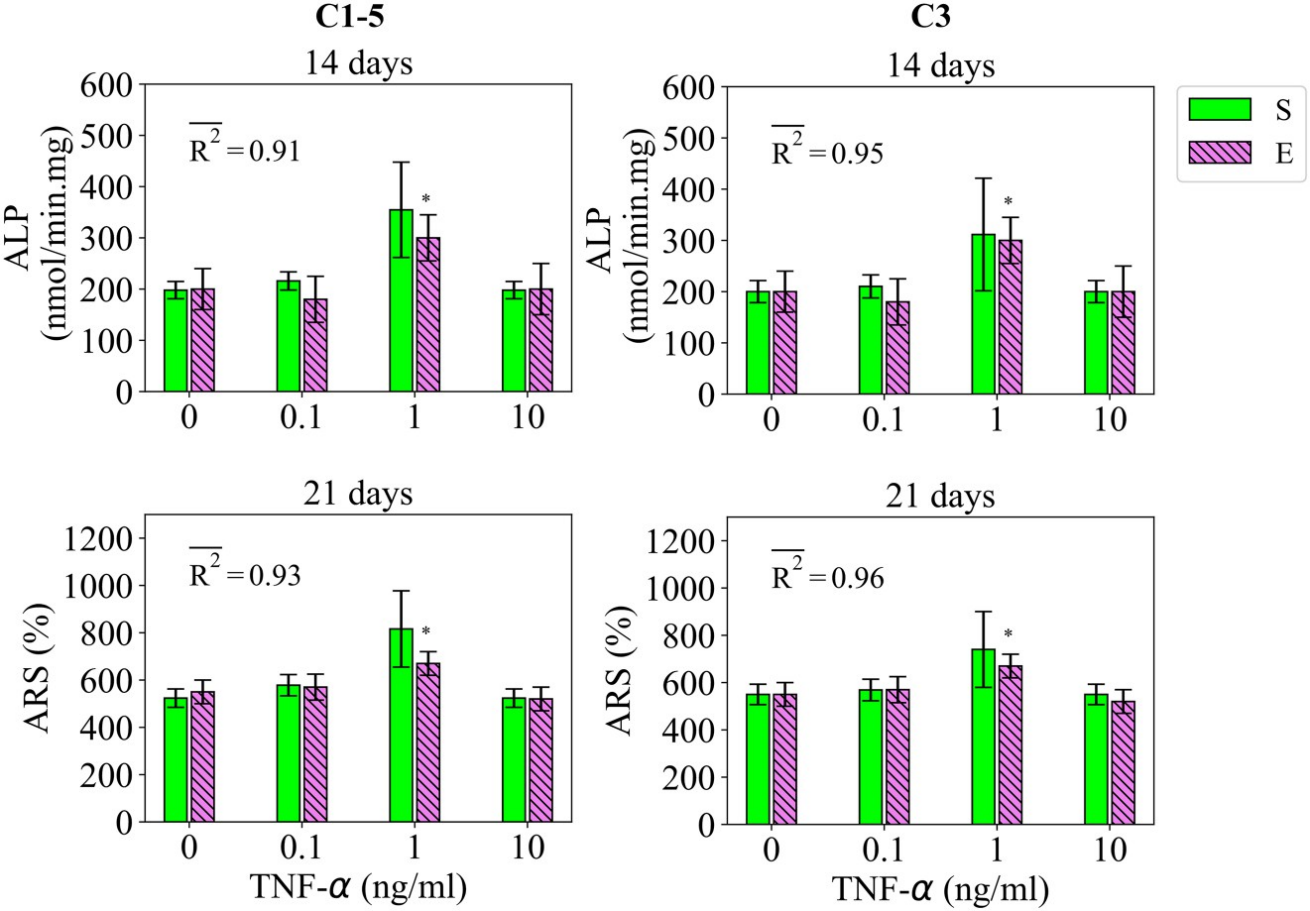
Fits of the model calibrated by C1-5 and C3 to the empirical data of study 3 for the case of TNF-α. Bars indicate the simulations (S) and the corresponding empirical data (E). The quantities of ALP and ARS are reported at day 14 and 21, respectively. The error bars on the empirical data shows the standard deviations. The error bars on the simulation results show the standard deviations obtained during SSIP, i.e. 15% alteration in the estimated parameter values. Stars indicate the statistically significant differences between values given for the empirical data compared to the control, i.e. the applied concentration of 0 ng/ml (p < 0.05 = *). $\overset{-}{R^{2}}$ is the average fitness value of the simulations for the given measurement item.

Study 3 reports the regulatory influence of IL-10 and TNF-α on the early and late differentiation rates, measured by ALP and ARS at day 14 and 21, respectively, for the application period of 48 hours [13]. The fits of the model to the data of study 3 are given in Fig 4 for IL-10 and in Fig 5 for TNF-α. In consistent with the empirical data, the model calibrated by C1-5 showed the stimulatory effect of IL-10 on both early and late differentiation rates within the concentration range of 0 to 10 ng/ml (see Fig 4). The obtained fitness values were 0.88 and 0.97 for ALP and ARS, respectively (see Fig 4). Once calibrated by C3, the fits of the model to the data experienced only a slight improvement (see Fig 4). The variations in the simulation results of study 3 due to SSIP are given in Fig 4 for the case of IL-10.

In agreement with the experiments, the model calibrated by C1-5 successfully captured the stimulatory effect of TNF-α on the differentiation rates at the applied concentration of 1ng/ml (see Fig 5). C1-5 produced the fitness values of 0.91 and 0.93 for ALP and ARS, respectively, for the case of TNF-α (see Fig 5). Once calibrated by C3, the fits of the model improved to 0.95 and 0.96 for ALP and ARS, respectively (see Fig 5). The variations in the simulation results of study 3 due to SSIP are given in Fig 5 for the case of TNF-α.

#### 2.2.4 Study 4

**Fig 6:**
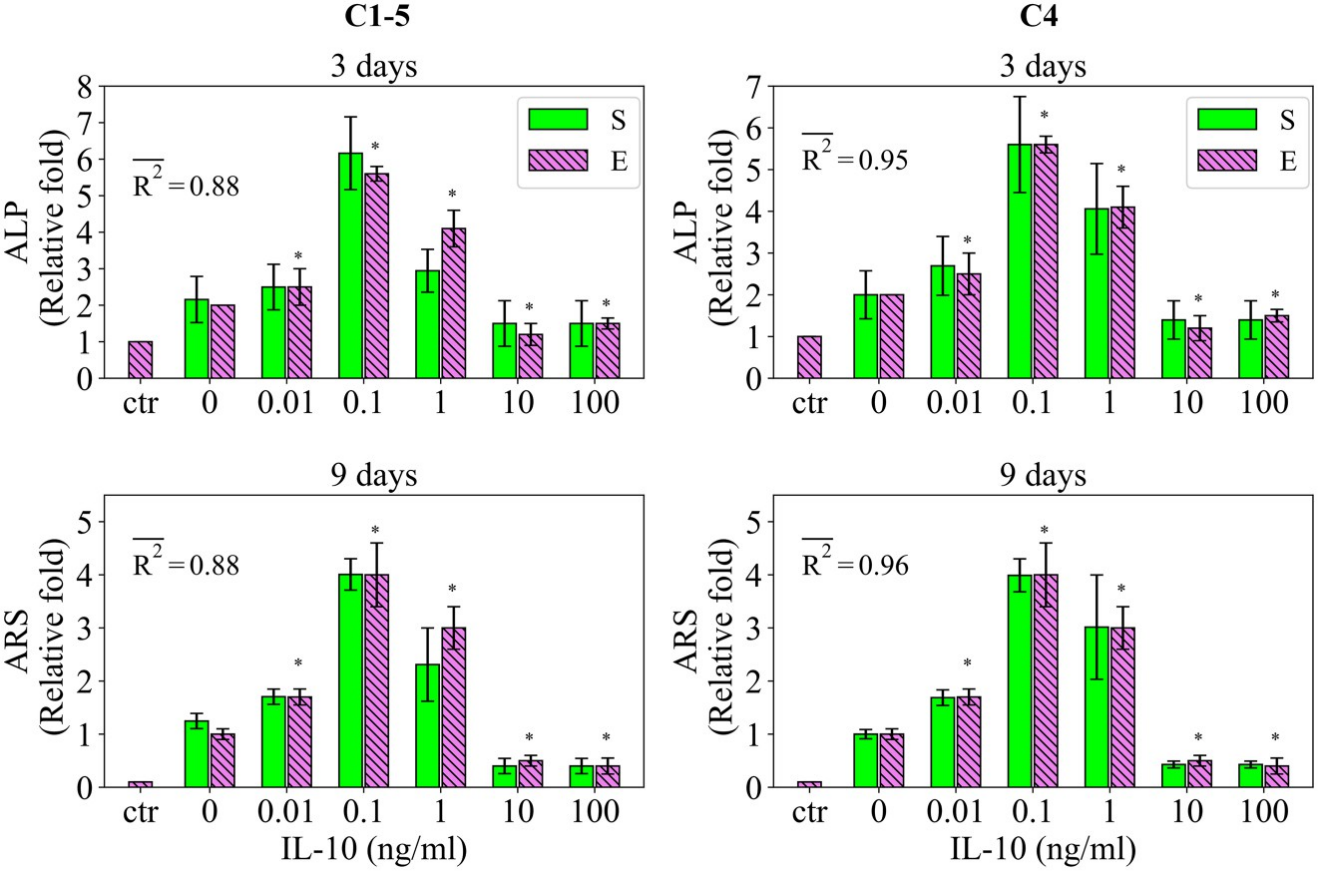
Fits of the model calibrated by C1-5 and C4 to the empirical data of study 4. Bars indicate the simulations (S) and the corresponding empirical data (E). The quantities of ALP and ARS are reported at day 3 and 9, respectively. The data is presented in a relative fold compared to the control, i.e. the undifferentiated case encoded as ctr. The error bars on the empirical data shows the standard deviations. The error bars on the simulation results show the standard deviations obtained during SSIP, i.e. 15% alteration in the estimated parameter values. Stars indicate the statistically significant differences between values given for the empirical data compared to the control (p < 0.05 = *). $\overset{-}{R^{2}}$ is the average fitness value of the simulations for the given measurement item.

Study 4 reports the effect of IL-10, administrated over 48 hours, on the early and late differentiation rates, measured by ALP and ARS, respectively [33]. The fits of the model to the data of study 4 are given in Fig 6. The data shows the stimulatory effect of IL-10 on the early and later differentiation rates once applied in the concentration range of 0 to 10 ng/ml with a maximum effect at the concentration of 0.1 ng/ml (see Fig 6). However, IL-10 shows an inhibitory effect on the differentiation process once applied at 10 ng/ml or more (see Fig 6). The model calibrated by C1-3 successfully captured these observations with the fitness values of 0.85 and 0.82 for ALP and ARS, respectively (see Fig 6). However, the simulation results notably deviate from the empirical data for the applied concentration of 1 ng/ml (see Fig 6). Once calibrated by C4, the simulation results were improved with the fitness values of 0.95 and 0.96 for ALP and ARS, respectively (see Fig 6). The variations in the simulation results of study 4 due to SSIP are given in Fig 6 for different measurement items.

#### 2.2.5 Study 5

**Fig 7:**
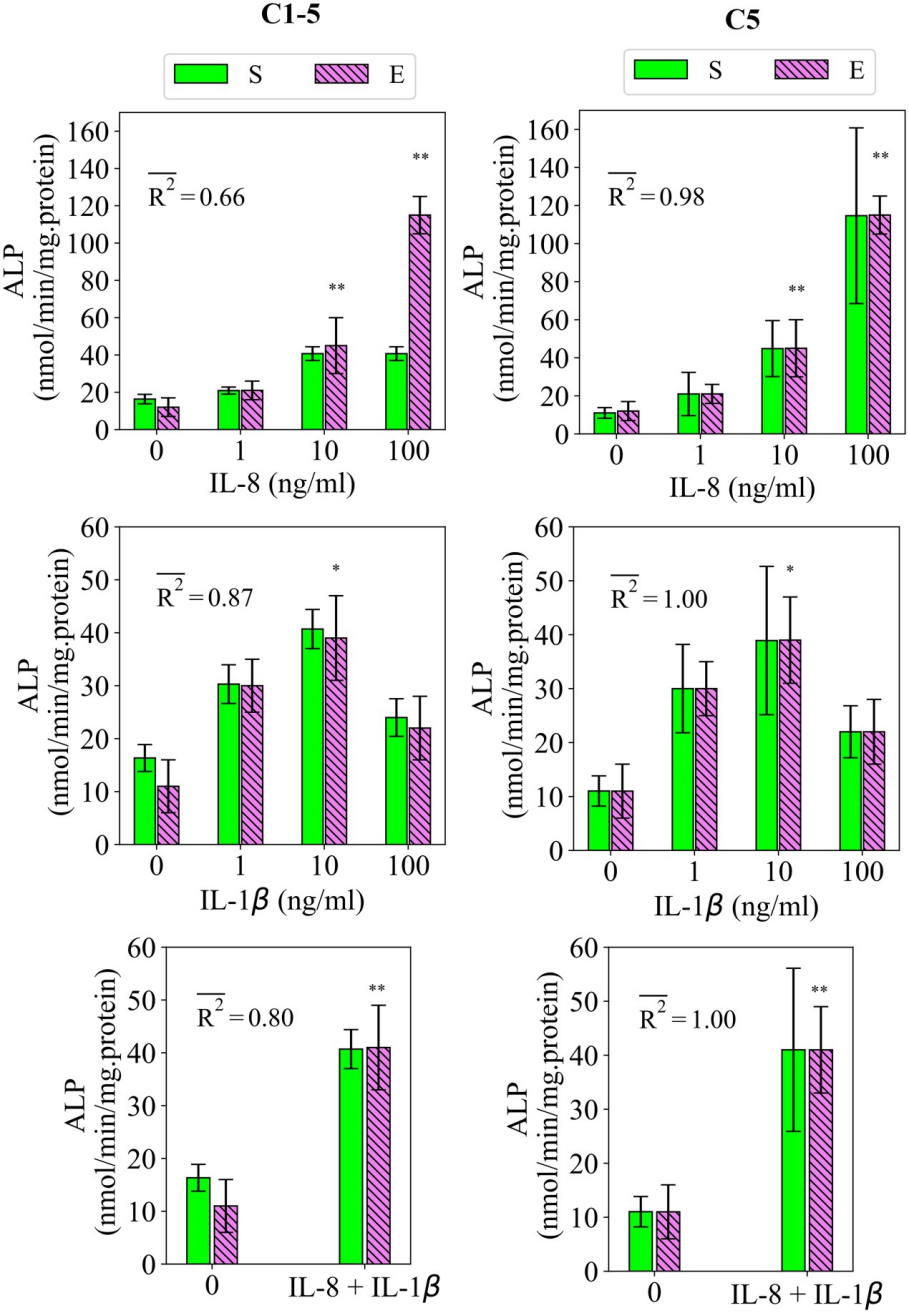
Fits of the model calibrated by C1-5 and C5 to the empirical data of study 5. Bars indicate the simulations (S) and the corresponding empirical data (E). The quantities of ALP are reported at day 9. The error bars on the empirical data shows the standard deviations. The error bars on the simulation results show the standard deviations obtained during SSIP, i.e. 15% alteration in the estimated parameter values. Stars indicate the statistically significant differences between values given for the empirical data compared to the control, i.e. the applied concentration of 0 ng/ml (p < 0.05 = *; p < 0.01 = **). $\overset{-}{R^{2}}$ is the average fitness value of the simulations for the given measurement item.

Study 5 measures the regulatory effects of IL-8 and IL-1β on the early differentiation rate, measured by ALP [17]. The fits of the model to the data of study 5 are presented in Fig 7. Based on the data, IL-8 stimulates the early differentiation rate in a dose-dependent fashion increasing from 0 to 100 ng/ml (see Fig 7). The model calibrated by C1-5 was not capable of reproducing the increase in ALP from 10 to 100 ng/ml (see Fig 7). Once calibrated by C5, the model reproduced the data throughout all applied concentrations. The fitness value was significantly improved from 0.66 to 0.98 from the case of C1-5 to C5 (see Fig 7). In the case of IL-1β, the empirical data show a significant stimulatory effect at the applied concentration of 10 ng/ml. The simulation results reproduced this observation with a fitness value of 0.87 (see Fig 7). Once calibrated by C5, the match of the simulation results to the data improved resulting in a fitness value of 1 (see Fig 7). By simultaneous application of IL-8 and IL-1β, the data showed a significant upregulation in the measured ALP (see Fig 7). The model calibrated by C1-5 reproduced this observation with a fitness value of 0.80 (see Fig 7). Once calibrated by C5, the simulation results perfectly matched the data with the fitness value of 1 (see Fig 7). The variations in the simulation results of study 5 due to SSIP are given in Fig 7 for different measurement items.

### 2.3 Results of the calibration process

**Fig 8:**
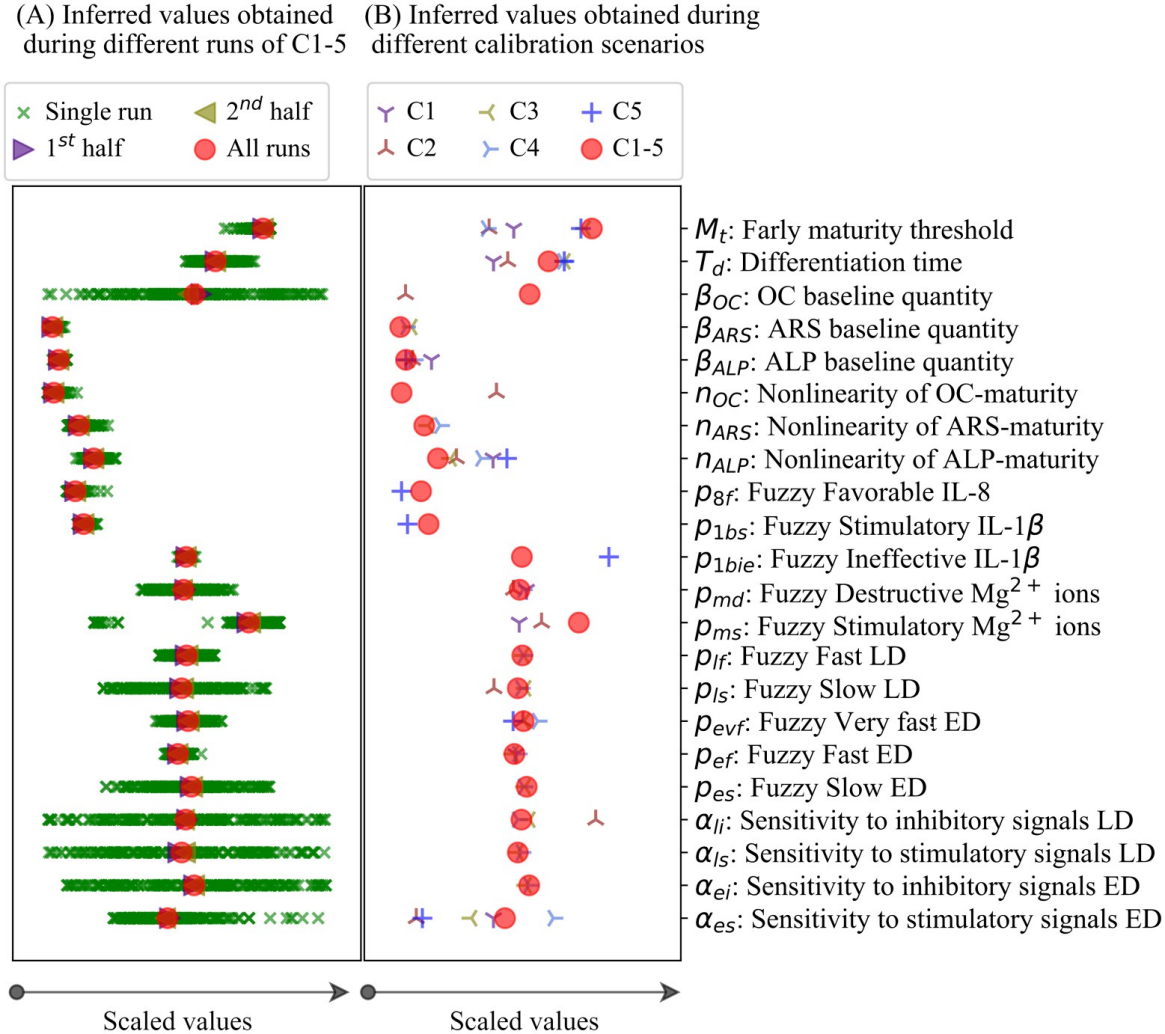
(A) Dispersity of the inferred values obtained during different runs of C1-5. In total, the calibration process is repeated 200 times in order to reach the stable inferred values, which is achieved by overlapping the mean values of all runs with the mean values of the 1^st^ and 2^nd^ halves of all runs. (B) Dispersity of the parameter values obtained during different calibration scenarios of C1, C2, C3, C4, C5, and C1-5. The values were scaled by dividing by the length of the priors. ED and LD stand for early differentiation and late differentiation, respectively.

The inferred parameter values obtained during different runs of C1-5 are given in Fig 8A. The results of the rest of the calibration scenarios, i.e. C1, C2, C3, C4, and C5 can be found in S1 Fig. The exact values of the inferred parameters are given in S3 Table. The results showed that different runs of the estimation process produce different sets of estimated values which can spread over the entire prior ranges (see Fig 8A and S1 Fig). It required 200, 200, 200, 200, 400, and 200 runs for C1, C2, C3, C4, C5, and C1-5, respectively, to reach consistent values for the inferred parameters. The inferred parameter values obtained during different calibration scenarios were plotted in Fig 8B in comparison with each other. The results showed that certain parameters are considerably different among different calibration scenarios. In particular, early maturation threshold (M_t_), differentiation time (T_d_), and the sensitivity of the early differentiation process to stimulatory signals (*α*_*es*_).

### 2.4 Results of the sensitivity analysis

The significance of the parameters obtained during different calibration scenarios is provided in Fig 3. It can be seen that LSSP and SSIP suggest different significance orders for the parameters. For instance, in the case of C5, *α*_*es*_ (sensitivity of the early differentiation rate to the stimulatory signals) is recognized as the top important parameter using SSIP, while this parameter is not among the top five parameters suggested by LSSP. Overall, the parameters of *n*_*ALP*_ and *β*_*ALP*_, which map the simulated maturity to the measured quantity of ALP in experiments, are among the first two significant parameters across different studies. In the second rank are the parameters of M_t_ (early maturity threshold), T_d_ (differentiation time), *p*_*ef*_ (the *Fast* membership level of early differentiation), and *α*_*es*_(sensitivity of the early differentiation rate to the stimulatory signals).

## 3. Discussion

### 3.1 The overall goodness of the model

**Fig 9:**
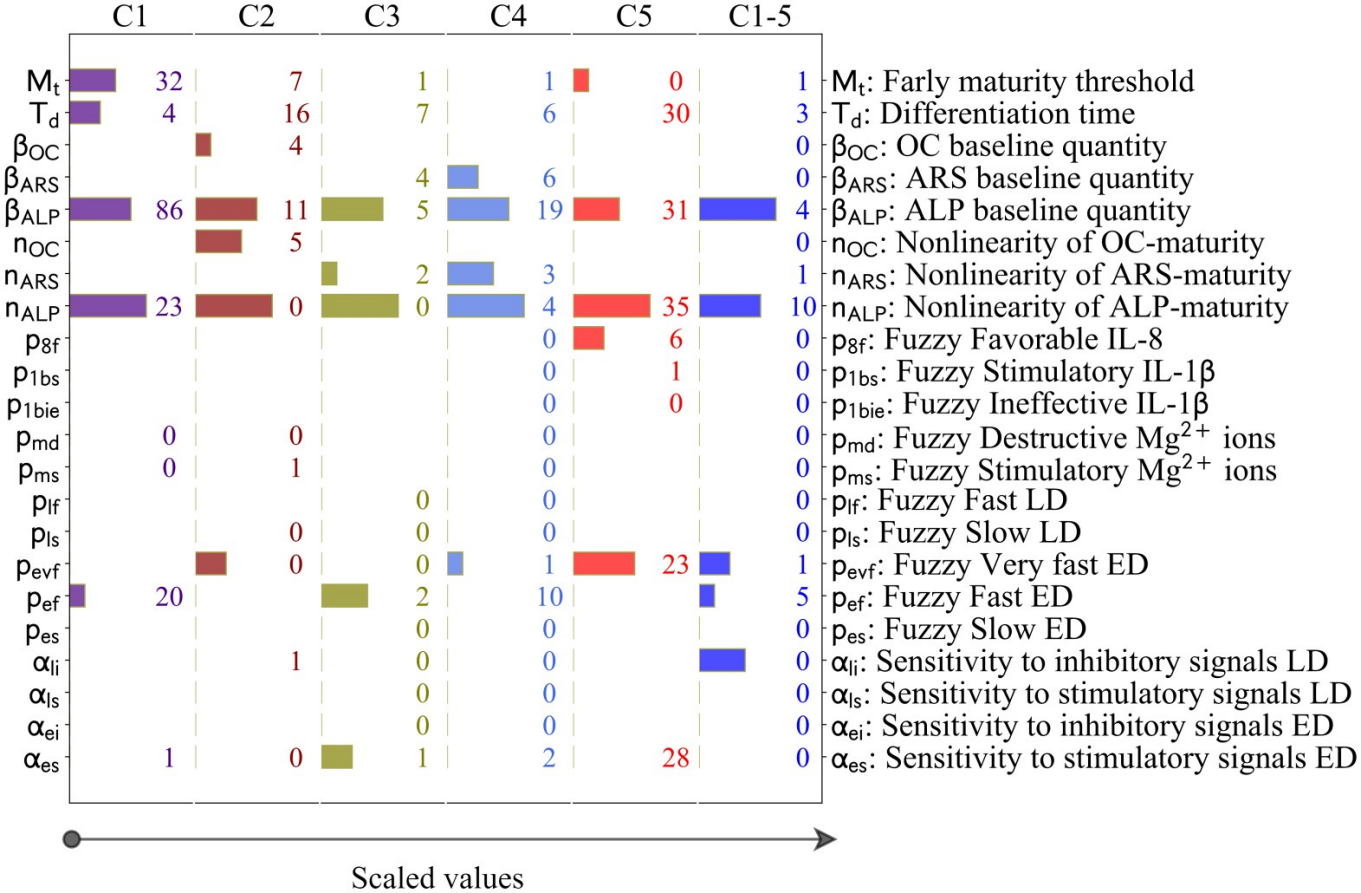
Results of the sensitivity analysis obtained during different calibration scenarios of C1, C2, C3, C4, C5 and C1-5. The bars indicate the five most significant parameters, given on the scale of 1 to 5, obtained from LSSP. The numbers show the results of SSIP in percentage. Those parameters with no values were not involved in that particular study.

In summary, the present model calibrated by the accumulated data of all studies, i.e. C1-5, was able to successfully reproduce most of the important empirical observations reported in studies 1 to 5 (Figs 2–7). In agreement with the experiments, the results of the simulations correctly demonstrated that Mg^2+^ ions in low concentration stimulates the early differentiation process, while at a concentration below the physiological level downregulates the differentiation phase (see Figs 2 and 3). The calibration process suggested 6–8 mM as the optimal concentration of Mg^2+^ ions in promoting the early differentiation rate (see the estimated value of p_ms_ in S3 Table). In addition, the model was capable of reproducing the following observations:

- The stimulatory effect of IL-10, once administrated for 48 hours, on the early and late differentiation processes within the concentration range of 0 to 10 ng/ml (see Fig 4)
- The stimulatory role of TNF-α once applied at the low concentration of 1 ng/ml (see Fig 5)
- The dose-dependent role of IL-10, once administrated over 48 hours, on early and late differentiation processes-an increasing trend in the stimulatory role within the concentration of 0 to 0.1 ng/ml and an inhibitory role at the concentration of above 1 ng/ml (see Fig 9)
- The increasing trend in the stimulatory effect of IL-8 on the early differentiation process within the concentration range of 0 to 10 ng/ml (see Fig 9)
- The stimulatory role of IL-1β on the early differentiation rate once applied at the concentration of 10 ng/ml (see Fig 9)
- The cumulative stimulatory effect of IL-8 and IL-1β on the early differentiation process once administrated together (see Fig 9).

However, the simulation results deviated from the experiments in capturing the inhibitory effect of Mg^2+^ ions on the late differentiation process (see Fig 3). In addition, the model was unable to reproduce the increasing trend in the stimulatory effect of IL-8 on the early differentiation rate once the applied concentration increased from 10 to 100 ng/ml (see Fig 7). Moreover, the simulation results were noticeably different from the empirical data for several observations; for instance, the quantity of ALP in study 3 for the applied concentration of 0.1 ng/ml (see Fig 4) and the quantity of ALP in study 4 for the applied concentration of 1 ng/ml (see Fig 6). In the next step, we conducted the calibration process for each study individually, i.e. C1, C2, C3, C4, and C5. The overall fits of the model to the empirical data improved significantly with an average accuracy of over 95 percent (see Figs 2–7).

### 3.2 Investigating the sources of discrepancy between the data and the simulation

In order to investigate the possible sources of discrepancy among the empirical data of different studies, we examined the estimated parameters obtained during different calibration scenarios (see Fig 8B). The results showed that the values of several parameters were noticeably different between different calibration schemes (see section 2.3). Such discrepancy can stem from either the different exploration of the calibration algorithm in finding the global minimums or the fundamental differences between different studies [31].

As previously noted, the model calibrated by C1-5 could not explain the inhibitory effect of Mg^2+^ ions on the late differentiation process, measured by OC in study 2 (see Fig 3). According to Fig 8B, the parameters of M_t_, T_d_, *β*_*OC*_, *n*_*OC*_, *α*_*li*_, and *α*_*es*_ show divergence between C1-5 and C2 (see Fig 2). *β*_*OC*_ and *n*_*OC*_, which map the simulated maturity to OC (see Eq (6)), are not associated with the calculation of the simulated maturity (see Eq (1) in section 4.4). Therefore, we exclude them from further evaluation. To examine the remaining parameters, we first elaborate on the regulatory effect of Mg^2+^ ions on the differentiation process. In the present model, the differentiation process is divided into the early and late phases (see Eq (1)). According to the fuzzy rules, Mg^2+^ ions stimulate the early differentiation rate while inhibiting the late differentiation rate (see Table 1). Therefore, the outcome of the differentiation progress is partly upregulated and partly downregulated by Mg^2+^ ions. M_t_ and T_d_ control the degree of contribution of each part in the final outcome of the differentiation process (see Eqs (1) and (2)). The lower quantity of M_t_, i.e. maturation threshold, obtained during C2 compared to C1-5 (see Fig 2B) shortens the early maturation phase and consequently constrains the stimulatory role of Mg^2+^ ions on the overall differentiation process. The lower quantity of T_d_, i.e. differentiation time, obtained during C2 compared to C1-5 (see Fig 2B) serves a similar purpose; a lower T_d_ results in an earlier maturation along the differentiation line, consequently limiting the stimulatory influence of Mg^2+^ ions. The evaluation of M_t_ and T_d_ suggests that the cells used in the experiments of study 2 possess a lesser differentiation capacity compared to the average of the rest of the studies.

**Table 1:** Fuzzy logic rules define the osteogenic reaction in response to stimulatory inputs. For the qualitative definition of each linguistic term, refer to Fig 10A. The symbol ⊕ indicates that the given conditions must occur simultaneously to produce the given intensity. The symbol ~ indicates that any choice of one or more from the chosen inputs produces the same intensity. The term ‘Not’, preceded by a linguistic level, indicates that the rule applied for all except the given level. The terms ‘ED’ and ‘LD’ stand for early differentiation and late differentiation, respectively. The rules are given in IF/THEN format in S2 Table in.

Cellular events		Cellular inputs				
Event	Intensity	TNF-α	IL-10	IL-8	IL-1β	Mg^2+^ ions
Early differentiation	Slow	~ Inhibitory	~ Inhibitory	-	-	~ Inhibitory ED or Destructive
	Physiological	⊕ Negligible or Ineffective	⊕ Negligible	⊕ Negligible	⊕ Negligible or Ineffective	⊕ Physiological or Ineffective
	Fast	~ Stimulatory	~ Favorable	-	-	~ Stimulatory
	Very fast	-	~ Stimulatory	~ ⊕ Favorable	~ ⊕ Negligible	-
				~ ⊕ Negligible	~ ⊕ Stimulatory	
				~ ⊕ Not Negligible	~ ⊕ Not Negligible	
	Extremely fast	-	-	⊕ Stimulatory	⊕ Negligible	-
Late differentiation	Slow	~ Inhibitory	~ Inhibitory	-	-	~ Inhibitory LD or Destructive
	Physiological	⊕ Negligible or Ineffective	⊕ Negligible	-	-	⊕ Not Inhibitory LD
	Fast	~ Stimulatory	~ Favorable	-	-	-
	Very fast	-	Stimulatory	-	-	-

Additionally, *α*_*es*_ and *α*_*li*_ contribute to the deviation of the model calibrated by C1-5 from the empirical data. *α*_*es*_ and *α*_*li*_ control the regulatory impact of Mg^2+^ ions on early and late differentiation rates, respectively (see Eq (3)). C2 reports a lower quantity of *α*_*es*_ and a higher quantity of *α*_*li*_, respectively, compared to C1-5 (see Fig 2B). These parameters, while possessing lesser significance on the simulation results compared to M_t_ and T_d_ (see Fig 9), contribute to reducing the overall effect of Mg^2+^ ions on the late differentiation process. This observation implies that cells used in the experiments of study 2 are more sensitive to stimulatory signals during early differentiation phase and less sensitive during late differentiation phase.

We previously noted that the model calibrated by C1-5 was not capable of capturing the increasing trend in the stimulatory effect of IL-8 on the early differentiation rate moving from 10 to 100 ng/ml, reported in study 5 (see Fig 7). According to Fig 8B, the values of the three parameters of *n*_*ALP*_, *p*_1*bie*_, and *α*_*es*_ were notably different between C1-5 and C5. As mentioned earlier, the parameter of *n*_*ALP*_ is not relevant in the calculation of the differentiation rate and is, therefore, excluded from further discussion. The parameter of *p*_1*bie*_, which is associated with IL-1β, is also irrelevant in the formulation of IL-8. Thus, we further examine the parameter of *α*_*es*_, i.e. the sensitivity of the differentiation process to the stimulatory factors, which is directly involved in the calculation of the early differentiation rate (see Eq (3)). It can be seen that C1-5 reports a higher quantity of *α*_*es*_ compared to C5 (see Fig 2B). According to Eq (3), a higher quantity of *α*_*es*_ increases the overall differentiation rate and results in a faster maturation process. Further evaluation of the model calibrated by C1-5 showed that the simulated maturity already reaches 1, i.e. ultimate value, for the applied concentration of 10 ng/ml, leaving no space for further improvement for higher stimulatory signals, e.g. 100 ng/ml (these results were not provided). This observation implies that the cells used in study 5 are less sensitive to stimulatory signals.

### 3.3 Calibration process

We showed that the employed DE approach results in different sets of parameter values at each run of the calibration process (see Fig 2A). In order to obtain consistent values, we repeated the tuning process at least two hundred times, depending on the study, and used the mean parameter values (see section 3.1). This approach is computationally expensive but results in consistency in the obtained parameter values, which can be used to investigate the differences among different models. Such discrepancy can be due to inherent differences among different experiments such as donor characteristics, culture medium characteristics, and experimental protocol [11], [17], [34].

### 3.4 Sensitivity analysis

In order to study the sensitivity of the model to its parameters, we employed two different approaches of LSSP and SSIP. LSSP captures the effect of large and simultaneous variations in the parameter values, while SSIP examines the impact of small and individual perturbations (see section 4.6). Together, these approaches provide a superior understanding of the system compared to the single approach utilized in our previous study [31]. In the formulation of the present model, we assumed non-linear relationships between the simulated maturity and the differentiation markers (see Eq (6)), which was assumed linear in our previous model [31]. The results of the sensitivity analysis showed *n*_*ALP*_, *n*_*ARS*_, and *n*_*OC*_, which control the degree of non-linearity in these relationships, are among the most significant parameters in the model (see Fig 9). The inferred values of these parameters were higher for the case of C1 to C5 compared to C1-5 (see Fig 8 and S3 Table). This is primarily due to the overfitting phenomenon as the calibrated models of C1 to C5 are too closely aligned to the data of individual studies. For the case of C1-5, the values of these parameters are close to 1 (see S3 Table), showing a linear relationship between the simulated maturity and the differentiation markers.

Another significant parameter in the model is *β*_*ALP*_, according to the results of the sensitivity analysis (see Fig 3). This parameter represents the baseline value of ALP secretion when the simulated maturity is zero (see Eq (6)). The estimated values of this parameter lie within 0.4 and 1.4 for different calibration scenarios (see S3 Table). The addition of this parameter to the relationship between the simulated maturity and the differentiation markers is an improvement to the formulation of our previous model, in which the baseline value is assumed zero [31]. The parameters of M_t_, T_d_, and *α*_*es*_ were also shown as the influential parameters according to the sensitivity analysis (see Fig 3). As elaborated earlier, these parameters, which are directly involved in the formulation of the simulated maturity, were the determinant factors in explaining the discrepancy in the simulation results.

### 3.5 Fuzzy-logic controller as the decision-making center of cell

In the present study, we used FL-based controllers as the deciding center of cells to simulate the process of osteogenic differentiation. FL-based simulations use plain language to describe a system which can potentially help in dissolving technical barriers between simulation and experimental experts easing their involvement in the rapid development of a computer model [26], [35]. Moreover, FL-based models are tractable and interpretable, which makes them a suitable option in investigating and incorporating the experimental datasets which are not in agreement with one another, similar to this study and our previous report [31]. Since the present model receives the signals from the environment and predicts cellular actions, similar to an agent, this descriptive model can serve as a natural basis for the predictive reinforcement model in the future [36].

### 3.6 Limitations, future developments, and concluding remarks

The proposed computer model in this study has several important limitations. Firstly, we were not able to find sufficient information in the literature to define the quality of interactions between different signaling factors in the FL controller, except for the two factors of IL-8 and IL-1β (see Table 1). A similar limitation was reported in our previous model [31] as well as in other computer models in the literature [37–39]. Further experiments are required to investigate the potential synergic effects between different cellular inputs, in particular, Mg^2+^ ions with the inflammatory cytokines [17]. Secondly, the proposed model in this study, which simulates the behavior of a single cell, is used to explain the average behavior of the cells cultured in the experiments. We defined several parameters to scale the outputs of the cell model to match the empirical observation (see section 4.5). However, the current model is not able to capture the heterogeneity in the behavior of individual cells. In the next step, the proposed cell model will be incorporated into an agent-based model to address this issue. This can potentially improve the simulation results but will require significantly higher computational costs, i.e. in the order of thousand times. Thirdly, the regulatory effect of substrate stiffness as an important factor in guiding osteogenesis is not incorporated in the present model [40]. This parameter will be included in our future studies. Similarly, transforming growth factor-beta (TGF-β) and bone morphogenic protein (BMP), which are two crucial growth factors in the osteogenic differentiation of MSCs, will be adopted from our previous model [31]. To do so, the quality of interaction between Mg^2+^ ions and these growth factors need to be studied first as Mg^2+^ ions are also reported to influence the activation of transforming growth factor-beta (TGF-β) and bone morphogenic protein (BMP) signaling [8]. Lastly, in this study, we simulated the influence of the inflammatory cytokines, primarily produced by macrophages, on MSC osteogenic differentiation. However, the cross-talk between MSC and macrophage is a double-sided process; macrophage polarization and cytokine production are also regulated by MSC. In the next steps, we will address this problem and complete the circle of MSC-macrophage interaction.

## 4 Materials and methods

**Fig 10:**
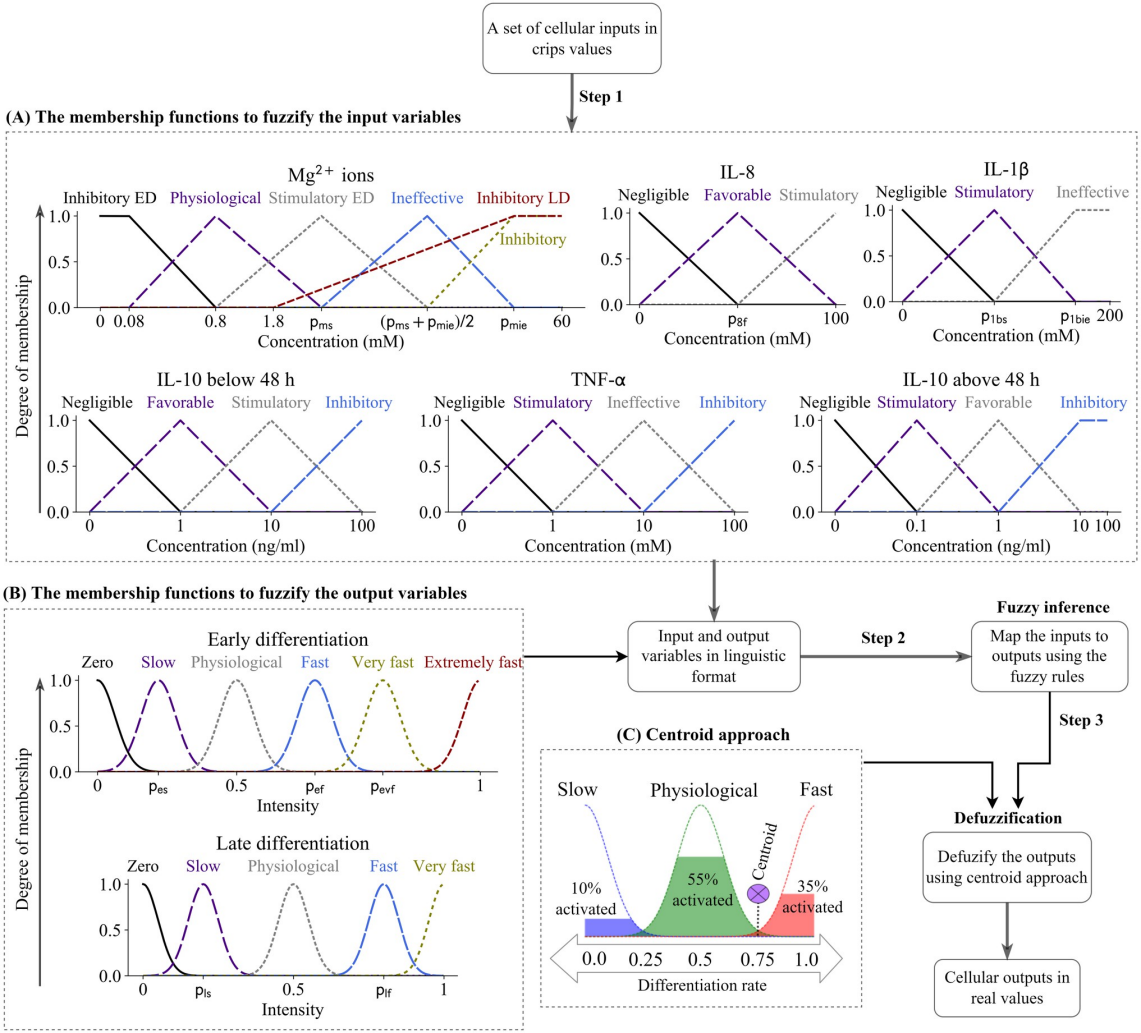
The complete flow of the implemented FL controller in this study. In step 1, the cellular inputs are transformed to linguistic variables using the membership functions given in (A). A set of triangular and trapezoid memberships functions are used during this fuzzification process. Similarly, cellular outputs of early and late differentiation rates are defined in linguistic formats using the membership functions given in (B). A set of Gaussian membership functions are used for this purpose. In step 2, the FL controller receives the cellular inputs and calculates the cellular outputs, both in linguistic form. In step 3, the outputs are defuzzified and converted into crisp, real values using the centroid approach (C). The terms ‘ED’ and ‘LD’ stand for early differentiation and late differentiation, respectively.

In this section, we first explain the complete process of the development of the FL controller (see Fig 10). The bioregulatory roles of the cellular inputs in the differentiation activities are explained in the following subsection together with the process of converting the qualitative knowledge into the machine-readable algorithm, termed fuzzification. After, the fuzzification process is explained for the cellular outputs. Then, the process of fuzzy inference and defuzzification is elaborated. The outputs of the FL controller require post-processing in order to define the actual rates of early and late differentiation rates. This process is further elaborated in the subsequent section. Finally, we elaborate on the empirical data used for the calibration and sensitivity analysis with the technical details of each process in the following sections. We used several software and packages to develop the current model, which can be found S1 Text. The source code of the present model can be found online [41].

### 4.1 Cellular inputs and fuzzification process

The first step in the development of the FL controller is to define and fuzzify the cellular inputs (see Fig 10). In this section, we elaborate on the bioregulatory roles of the five signaling factors in the early and late differentiation processes using the information available in the literature. Then, FL membership functions are defined according to this information.

**Mg**^**2+**^ **ions** are shown to affect osteogenic differentiation depending on the concentration and the state of cell differentiation [42–45]. A Mg^2+^ ion concentration of 0.8 mM, which is commonly used as the minimal essential Medium-MEM in cell culture experiments, is considered as the physiological concentration. A concentration below 0.8 mM is shown to delay the early differentiation process of MSCs [17]. Mg^2+^ ions within the concentration range of 2–10 mM is reported to promote early differentiation rate [6], [43], [45–47], while the concentration of Mg^2+^ ions above 1.8 mM is reported inhibitory for late differentiation process [6], [43], [45]–[47]. In addition, a concentration over 20 mM is shown to compromise cell viability [47–49]. To account for these observations, we create six membership functions to define the cellular input of Mg^2+^ ions as shown in Fig 10A. The parameter of *p*_*ms*_ is defined to mark the peak occurrence of the *Stimulatory* effect. The parameter of *p*_*md*_ marks the beginning of the *Inhibitory* effect. The membership function of *Ineffective* is defined as the transition state from *Stimulatory* to *Inhibitory* state.

**IL-10** is a key anti-inflammatory cytokine in the bone regenerative process including osteogenic differentiation [33]. The mediatory effects of IL-10 are shown to significantly depend on the applied concentration as well as the length of application [13], [33][50]. Chen et al. [33] showed that IL-10 has a dual effect on osteogenic activities; IL 10 in low concentration activates p38/MAPK signaling pathway and stimulates osteogenic activities, while higher concentrations of IL-10 inhibit p38/MAPK signaling by activating NF-kB and consequently downregulating osteogenesis. They found that IL-10 has the highest stimulatory effect at the concentration of 0.1 ng/ml, while the concentration of 10 ng/ml showed a significantly inhibitory effect on osteogenesis [33]. However, Valles et al. [13] showed an increasing stimulatory effect of IL-10 on osteogenesis by increasing the concentration from 0.1 to 10 ng/ml. They also experimented the importance of application time and concluded that while IL-10 can be pro-osteogenic after short-term treatment (48 hours), its continuous application can produce inhibitory effects [13]. Therefore, in the definition of the FL controller, we set the application time of 48 hours to differentiate short- and long- term treatment periods. Accordingly, we define two separate sets of functions to fuzzify the cellular input of IL-10 depending on the application time. In the controller that simulates IL-10 for the application of less or equal to 48 hours, we define a constant increase in the stimulatory effect of IL-10 from 0 to 10 ng/ml (see Fig 10A). However, for the case where the application time exceeds 48 hours, the promotory effect of IL-10 decreases by exceeding 0.1 ng/ml (see Fig 10A), in compliance with the findings of Chen et al. [33].

**TNF-α** is a primary cytokine in the inflammatory reaction which plays an important role in osteogenic differentiation [51]. The regulatory effects of TNF-α on osteogenesis have been shown to be concentration-dependent; while low concentration of TNF-α is assumed stimulatory, its high concentration has an inhibitory effect on osteoblastic differentiation [13], [52]. According to the results of Valles et al. [13] and Glass et al. [52], we define the stimulatory concentration of TNF-α as 1 ng/ml and the inhibitory concentration as 100 ng/ml [52]. TNF-α at a concentration of 10 ng/ml is shown to have neither stimulatory nor inhibitory effect on osteogenesis [13], [52]. Thus, the concentration of 10 ng/ml is defined as the neutral state with an ineffective role in osteogenesis (see Fig 10A).

**IL-8** is traditionally classified as a pro-inflammatory cytokine with the main role of recruiting the inflammatory cells to the injury site [17][53]. In addition, IL-8 is shown to play an important role in the commitment of MSCs to bone cells [54][55]. Qiao et al. [17] showed that IL-8 is especially important when the inflammatory response is mediated by Mg^2+^ ions; IL-8 is the primary player in promoting osteogenesis when macrophages are treated by Mg^2+^ ions [17]. They also showed that the regulatory effect of IL-8 on osteogenesis is dose-dependent and substantially increases within the concentration of 0 to 100 ng/ml [17]. To account for these observations, we define three membership functions to fuzzify the cellular input of IL-8 (see Fig 10A), where the stimulatory role of IL-8 rapidly increases by an increase in its concentration. The parameter of *p*_8*f*_ marks the peak occurrence of *Favorable* condition which is an intermediate state toward *Stimulatory* level.

**IL-1β** is a major pro-inflammatory cytokine with an important role in the simulation of osteoclastogenesis [56]. IL-1β is also shown to increase osteogenic activities of MSCs, especially when it is administrated in a low concentration within 1 to 10 ng/ml [17]. To account for these observations, we define three membership functions to fuzzify IL-1β (see Fig 10A), where two parameters of *p*_1*bs*_ and *p*_1*bie*_ mark the peak and end of the stimulatory effect. In addition, IL-1β is reported to be an antagonizer of IL-8 osteogenic effects [17]; Qiao et al. [17] showed that IL-1β substantially reduces the secretion of the osteogenic biomarkers stimulated by IL-8. Thus, in the present FL controller, IL-1β has a dual role in osteogenetic response; on the one hand, it stimulates osteogenesis; on the other hand, it hampers the stimulatory role of IL-8.

### 4.2 Cellular outputs and fuzzification process

The outputs of the fuzzy controller are early and late differentiation rates. These values are defined in linguistic formats using the set of membership functions given in Fig 10B. The early differentiation process is assumed to occur in six different rates within the range of 0 and 1, with 0.5 representing the physiological rate. The parameters of *p*_*es*_, *p*_*ef*_, and *p*_*evf*_ are defined to mark the intensities of *Slow*, *Fast*, and *Very fast* levels, accordingly. The late differentiation process is fuzzified using five membership functions shown in Fig 10B, with 0.5 representing the physiological rate (see Fig 10B). The parameter of *p*_*ls*_ and *p*_*lf*_ are defined to mark the *Slow* and *Fast* membership levels. Gaussian membership functions (GMFs) with the activation value of 1 and the sigma value of 0.05 are employed for the fuzzification of the output variables. GMFs offer multiple advantageous such as smoothness and concise notation as well as superior reliability and robustness of the system [57][58].

### 4.3 Fuzzy inference and defuzzification process

The set of rules given in Table 1 is used to determine the differentiation rates in response to the cellular inputs. A Mamdani-type FL controller is used for the calculations. Since multiple rules can be triggered simultaneously, the centroid technique is used to determine the final output of the controller. In this technique, the area bound by the activation degree is summed over different fuzzy sets, and the center of the sums is calculated to represent the final outcome (see Fig 10C) [59].

### 4.4 Simulation of the differentiation process

Similar to our previous study [31], we define the factor of *maturity* to mark the degree of progression along the line of osteogenic differentiation. *Maturity* holds a value in the range of 0 and 1 and is calculated for a given time (T) using early and late differentiation rates (r_*e*_ and r_*l*_, respectively) as,

$$Maturity=\left\{\begin{array}{c} T\cdot r_{e},\;T\leq T_{e}\\ T_{e}\cdot r_{e}+\left(T-T_{e}\right)\cdot r_{l},\;T>T_{e} \end{array}\right.$$ (1)

where *T*_*e*_ is time required for the early maturation process.

$$T_{e}={\text{M}}_{t}\cdot T_{d}$$ (2)

where M_*t*_ is the early maturation threshold, and T_d_ is the time required for MSC to fully differentiate to osteoblast. M_*t*_ is a value between 0 and 1, marking the end of early differentiation process.

In Eq (1), r_*e*_ and r_*l*_ are the scaled versions of *f*_*e*_ and *f*_*l*_ (fuzzy outputs), respectively,

$${\text{r}}_{e}={\text{r}}_{0}\cdot S(f_{e})$$ (3)

$${\text{r}}_{l}={\text{r}}_{0}\cdot S(f_{l})$$ (4)

where r_0_ is the physiological rate of osteogenic differentiation, calculated as 1/T_d_, where S is a function that scales the outputs of fuzzy controller, which are initially between 0 and 1,

$$S\left(x\right)=\left\{\begin{array}{c} \;2\cdot \alpha_{s,k}\cdot \left(\text{x}-0.5\right)+1,\;x\leq 0.5\\ 2\cdot \alpha_{i,k}\cdot \left(\text{x}-0.5\right)+1,\;x>0.5 \end{array}\right.$$ (5)

where x is the input from the FL controller. α_*s*,*k*_ and α_*i*,*k*_ are the scaling coefficients for the stimulatory and inhibitory effects, respectively, *k* ∈ {*e*, *f*}.

### 4.5 Empirical data

The empirical data obtained from 5 sets of published experiments are used to estimate the free parameters of the present model (see S1 Table for the summary of the experiments). These experiments evaluate the bioregulatory effect of different Mg^2+^ ions as well as different inflammatory cytokines on the osteogenic differentiation of MSCs. Three markers of ALP, OC, and ARS are used to study the differentiation process. ALP is commonly used as an early differentiation marker, while OC and ARS are recognized as the markers of the late differentiation phase [40], [60]. These factors are correlated with *maturity*, which is the simulated indicator of differentiation progress in the present study,

$$y_{i}\left(\text{x}\right)={\left(x\;+\beta_{i}\right)}^{n_{i}}\cdot k_{i,j}$$ (6)

where x is the conditioned value of maturity, *y*_*i*_ is the quantity of the biomarker with i ∈ {*ALP*, *OC*, *ARS*}, β_*i*_ is the baseline of the marker, *n*_*i*_ is the degree of nonlinearity, and *k*_*i*,*j*_ is the correction factor applied for each experiment with *j* ∈ 1:5. For ALP, x is calculated as,

$$x=\left\{\begin{array}{c} maturity,\;maturity\leq M_{t}\\ \;M_{t},\;maturity>M_{t} \end{array}\right.$$ (7)

In Eq (7), we assume that ALP correlates with maturity until the early maturation threshold and stays constant afterward, accounting for the fact that ALP is the early differentiation marker. OC and ARS, as the late marker of differentiation, correlate with maturity in its whole range, i.e. x = maturity. In Eq (6), β_*i*_ takes into account that the differentiation markers can be detected even at the beginning of an experiment where the simulated maturity is zero [33]. The parameter of n_*i*_ is defined to catch the non-linear correlation between maturity and the differentiation markers. Lastly, k_*i*,*j*_ takes into account the variations in the measurements of the markers across different experiments, e.g. differences in the reported units.

### 4.6 Calibration process and sensitivity analysis

**Fig 11:**
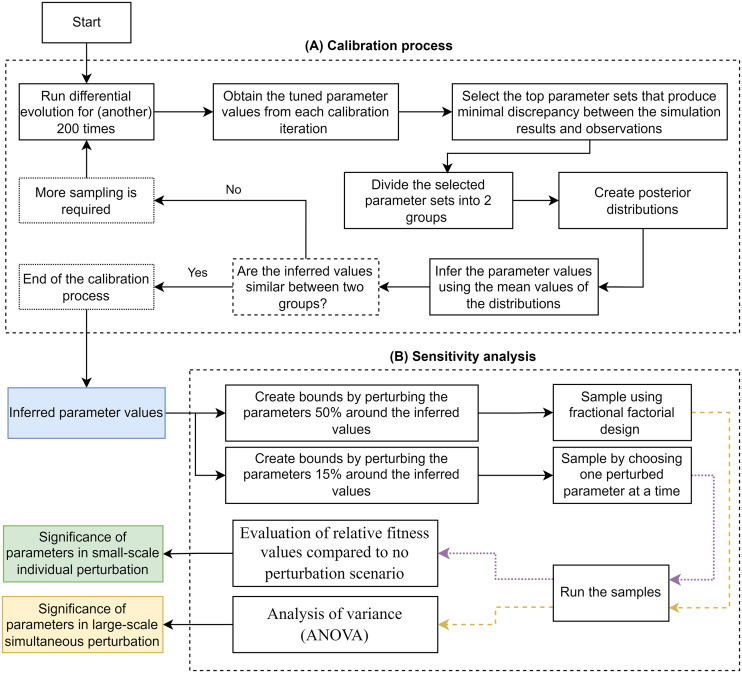
The complete flow of the calibration process and the sensitivity analysis implemenetd in this study.

The overview of the calibration process is given in Fig 11A. DE is a metaheuristic stochastic search algorithm based on an evolutionary process which improves the candidate solution by an iterative approach [61]. Although DE is considered a global optimization algorithm, the estimated parameter values can be different at each run. To overcome this issue, we execute multiple DE runs and use the posterior distributions to infer the parameter values (see Fig 11A).

The overview of the sensitivity analysis is given in Fig 11B. Two approaches of LSSP and SSIP are employed to study the sensitivity of the model to its parameters. In LSSP, one or several parameters are simultaneously perturbed with a magnitude of 50 percent around the inferred values. Fractional factorial design (FFD) and the analysis of variance (ANOVA) are used for sampling and analysis of this approach, which are explained in detail in our previous publication [31]. In SSIP, the parameters of the model are perturbed one at a time, with a magnitude of 15 percent around the inferred values.

## Supporting information

S1 Fig Dispersity of the parameter values obtained during the calibration process for different calibration scenarios of C1, C2, C3, C4, C5 and C1-5. Individual runs are the results of each calibration process; All samples represent the mean of combined individual runs; First (1st) and second (2nd) halfs of samples indicate the means of the frist half and second half of combined individual runs, respectively. The values were scaled by dividing by the length of the priors.
(TIFF)

S1 Table The summary of the specifications of the cell culture experiments used to estimate the model’s free parameters. (DOCX)

S2 Table The fuzzy logic rules in IF/THEN format. The words in green are the cellular inputs while those in red are cellular outputs.
(DOCX)

S3 Table The list of the free parameters in the present model, and the inferred values during different calibration schemes. Those marked by ‘-‘were not inferred during that particular calibrations scenario.
(DOCX)

S1 Text Specification of the software used in this study. (DOCX)

## References

[1] Willumeit-Römer R. The Interface Between Degradable Mg and Tissue. Jom. 2019;71(4):1447–55.

[2] Rahmati M, Silva EA, Reseland JE, Heyward CA., Haugen HJ. Biological responses to physicochemical properties of biomaterial surface. Chem Soc Rev. 2020;49(15):5178–224. doi: 10.1039/d0cs00103a 32642749

[3] Zhang J, Ma X, Lin D, Shi H, Yuan Y, Tang W, et al. Magnesium modification of a calcium phosphate cement alters bone marrow stromal cell behavior via an integrin-mediated mechanism. Biomaterials. 2015;53:251–64. doi: 10.1016/j.biomaterials.2015.02.097 25890724

[4] Qi T, Weng J, Yu F, Zhang W, Li G, Qin H, et al. Insights into the Role of Magnesium Ions in Affecting Osteogenic Differentiation of Mesenchymal Stem Cells. Biol Trace Elem Res. 2021;199(2):559–67. doi: 10.1007/s12011-020-02183-y 32449009

[5] Lin S, Yang G, Jiang F, Zhou M, Yin S, Tang Y, et al. A magnesium-enriched 3D culture system that mimics the bone development microenvironment for vascularized bone regeneration. Adv Sci. 2019;6(12):1900209. doi: 10.1002/advs.201900209 31380166 PMC6662069

[6] Luthringer BJC, Willumeit-Römer R. Effects of magnesium degradation products on mesenchymal stem cell fate and osteoblastogenesis. Gene [Internet]. 2016;575(1):9–20. Available from: doi: 10.1016/j.gene.2015.08.028 26283150

[7] Wang Y, Geng Z, Huang Y, Jia Z, Cui Z, Li Z, et al. Unraveling the osteogenesis of magnesium by the activity of osteoblasts in vitro. J Mater Chem B. 2018;6(41):6615–21. doi: 10.1039/c8tb01746h 32254870

[8] Li RW, Kirkland NT, Truong J, Wang J, Smith PN, Birbilis N, et al. The influence of biodegradable magnesium alloys on the osteogenic differentiation of human mesenchymal stem cells. J Biomed Mater Res Part A. 2014;102(12):4346–57. doi: 10.1002/jbm.a.35111 24677428

[9] Zhang X, Chen Q, Mao X. Magnesium Enhances Osteogenesis of BMSCs by Tuning Osteoimmunomodulation. Biomed Res Int. 2019;2019. doi: 10.1155/2019/7908205 31828131 PMC6885163

[10] Díez-Tercero L, Delgado LM, Bosch-Rué E, Perez RA. Evaluation of the immunomodulatory effects of cobalt, copper and magnesium ions in a pro inflammatory environment. Sci Rep [Internet]. 2021;11(1):1–13. Available from: doi: 10.1038/s41598-021-91070-0 34083604 PMC8175577

[11] Zhang Y, Böse T, Unger RE, Jansen JA, Kirkpatrick CJ, van den Beucken JJJP. Macrophage type modulates osteogenic differentiation of adipose tissue MSCs. Cell Tissue Res. 2017;369(2):273–86. doi: 10.1007/s00441-017-2598-8 28361303 PMC5552848

[12] Carty F, Mahon BP, English K. The influence of macrophages on mesenchymal stromal cell therapy: Passive or aggressive agents. Clin Exp Immunol. 2017;188(1):1–11. doi: 10.1111/cei.12929 28108980 PMC5343357

[13] Valles G, Bensiamar F, Maestro-Paramio L, García-Rey E, Vilaboa N, Saldaña L. Influence of inflammatory conditions provided by macrophages on osteogenic ability of mesenchymal stem cells. Stem Cell Res Ther. 2020;11(1):1–15. 32054534 10.1186/s13287-020-1578-1 PMC7020593

[14] Gong L, Zhao Y, Zhang Y, Ruan Z. The macrophage polarization regulates MSC osteoblast differentiation in vitro. Ann Clin Lab Sci. 2016;46(1):65–71. 26927345

[15] Romero-López M, Li Z, Rhee C, Maruyama M, Pajarinen J, O’Donnell B, et al. Macrophage effects on mesenchymal stem cell osteogenesis in a three-dimensional in vitro bone model. Tissue Eng—Part A. 2020;26(19–20):1099–111. doi: 10.1089/ten.TEA.2020.0041 32312178 PMC7580572

[16] Mazur A, Maier JAM, Rock E, Gueux E, Nowacki W, Rayssiguier Y. Magnesium and the inflammatory response: potential physiopathological implications. Arch Biochem Biophys. 2007;458(1):48–56. doi: 10.1016/j.abb.2006.03.031 16712775

[17] Qiao W, Wong KHM, Shen J, Wang W, Wu J, Li J, et al. TRPM7 kinase-mediated immunomodulation in macrophage plays a central role in magnesium ion- induced bone regeneration. Nat Commun [Internet]. 2021;12(2885). Available from: doi: 10.1038/s41467-021-23005-2 34001887 PMC8128914

[18] Leidi M, Dellera F, Mariotti M, Maier JAM. High magnesium inhibits human osteoblast differentiation in vitro. Magnes Res. 2011;24(1):1–6. doi: 10.1684/mrh.2011.0271 21421455

[19] Zhou H, Liang B, Jiang H, Deng Z, Yu K. Magnesium-based biomaterials as emerging agents for bone repair and regeneration: from mechanism to application. J Magnes Alloy. 2021;(xxxx).

[20] da Silva Lima F, da Rocha Romero AB, Hastreiter A, Nogueira-Pedro A, Makiyama E, Colli C, et al. An insight into the role of magnesium in the immunomodulatory properties of mesenchymal stem cells. J Nutr Biochem [Internet]. 2018;55:200–8. Available from: doi: 10.1016/j.jnutbio.2018.02.006 29554498

[21] Metzcar J, Wang Y, Heiland R, Macklin P. A Review of Cell-Based Computational Modeling in Cancer Biology. JCO Clin Cancer Informatics. 2019 Feb;(3):1–13. doi: 10.1200/CCI.18.00069 30715927 PMC6584763

[22] Prasad A, Alizadeh E. Cell Form and Function: Interpreting and Controlling the Shape of Adherent Cells. Vol. 37, Trends in Biotechnology. Elsevier Ltd; 2019. p. 347–57. 10.1016/j.tibtech.2018.09.007 30316557

[23] Nourisa J, Rouhi G. Prediction of the trend of bone fracture healing based on the results of the early stages simulations: a finite element study. J Mech Med Biol. 2019;19(5).

[24] Nourisa J, Rouhi G. Biomechanical evaluation of intramedullary nail and bone plate for the fixation of distal metaphyseal fractures. J Mech Behav Biomed Mater. 2016;56. doi: 10.1016/j.jmbbm.2015.10.029 26655955

[25] Zhao C, Medeiros TX, Sové RJ, Annex BH, Popel AS. A data-driven computational model enables integrative and mechanistic characterization of dynamic macrophage polarization. iScience. 2021;102112. doi: 10.1016/j.isci.2021.102112 33659877 PMC7895754

[26] Bordon J, Moskon M, Zimic N, Mraz M. Fuzzy Logic as a Computational Tool for Quantitative Modelling of Biological Systems with Uncertain Kinetic Data. IEEE/ACM Trans Comput Biol Bioinforma. 2015;12(5):1199–205. doi: 10.1109/TCBB.2015.2424424 26451831

[27] Nobile MS, Votta G, Palorini R, Spolaor S, De Vitto H, Cazzaniga P, et al. Fuzzy modeling and global optimization to predict novel therapeutic targets in cancer cells. Bioinformatics [Internet]. 2019;36(7):2181–8. Available from: doi: 10.1093/bioinformatics/btz868 31750879 PMC7141866

[28] Aldridge BB, Saez-Rodriguez J, Muhlich JL, Sorger PK, Lauffenburger DA. Fuzzy Logic Analysis of Kinase Pathway Crosstalk in TNF/EGF/Insulin-Induced Signaling. PLoS Comput Biol. 2009;5(4). doi: 10.1371/journal.pcbi.1000340 19343194 PMC2663056

[29] Wang M, Yang N. Three-dimensional computational model simulating the fracture healing process with both biphasic poroelastic finite element analysis and fuzzy logic control. Sci Rep [Internet]. 2018;8(1):1–13. Available from: doi: 10.1038/s41598-018-25229-7 29712979 PMC5928059

[30] Niemeyer F, Claes L, Ignatius A, Meyers N, Simon U. Simulating lateral distraction osteogenesis. PLoS One. 2018;13(3):e0194500. doi: 10.1371/journal.pone.0194500 29543908 PMC5854389

[31] Nourisa J, Zeller-Plumhoff B, Helmholz H, Luthringer-Feyerabend B, Ivannikov V, Willumeit-Römer R. Magnesium ions regulate mesenchymal stem cells population and osteogenic differentiation: a fuzzy agent-based modeling approach. Comput Struct Biotechnol J [Internet]. 2021;19:4110–22. Available from: doi: 10.1016/j.csbj.2021.07.005 34527185 PMC8346546

[32] Price K V. Differential evolution. In: Handbook of optimization. Springer; 2013. p. 187–214.

[33] Chen E, Liu G, Zhou X, Zhang W, Wang C, Hu D, et al. Concentration-dependent, dual roles of IL-10 in the osteogenesis of human BMSCs via P38/MAPK and NF-kB signaling pathways. FASEB J. 2018;32(9):4917–29. 29630408 10.1096/fj.201701256RRR

[34] Sarugaser R, Lickorish D, Baksh D, Hosseini MM, Davies JE. Human umbilical cord perivascular (HUCPV) cells: a source of mesenchymal progenitors. Stem Cells. 2005 Feb;23(2):220–9. doi: 10.1634/stemcells.2004-0166 15671145

[35] Spolaor S, Gribaudo M, Iacono M, Kadavy T, Oplatková ZK, Mauri G, et al. Towards human cell simulation. In: Lecture Notes in Computer Science (including subseries Lecture Notes in Artificial Intelligence and Lecture Notes in Bioinformatics). Springer Verlag; 2019. p. 221–49.

[36] Esteva A, Robicquet A, Ramsundar B, Kuleshov V, DePristo M, Chou K, et al. A guide to deep learning in healthcare. Vol. 25, Nature Medicine. Nature Publishing Group; 2019. p. 24–9. 10.1038/s41591-018-0316-z 30617335

[37] Geris L, Gerisch A, Vander Sloten J, Weiner R, Van Oosterwyck H. Angiogenesis in bone fracture healing: A bioregulatory model. J Theor Biol. 2008;251(1):137–58. doi: 10.1016/j.jtbi.2007.11.008 18155732

[38] Zhao C, Mirando AC, Sové RJ, Medeiros TX, Annex BH, Popel AS. A mechanistic integrative computational model of macrophage polarization: Implications in human pathophysiology. PLoS Comput Biol. 2019;15(11):1–28. doi: 10.1371/journal.pcbi.1007468 31738746 PMC6860420

[39] Kuhn C, Checa S. Computational modeling to quantify the contributions of VEGFR1, VEGFR2, and lateral inhibition in sprouting angiogenesis. Front Physiol. 2019;10(MAR):1–14. 30971939 10.3389/fphys.2019.00288 PMC6445957

[40] Bahney CS, Zondervan RL, Allison P, Theologis A, Ashley JW, Ahn J, et al. Cellular biology of fracture healing. J Orthop Res. 2019;37(1):35–50. doi: 10.1002/jor.24170 30370699 PMC6542569

[41] Nourisa J. janursa/MSC_OB_Mg_ICs: [Internet]. Zenodo; 2022.

[42] Wu L, Feyerabend F, Schilling AF, Willumeit-Romer R, Luthringer BJ. Effects of extracellular magnesium extract on the proliferation and differentiation of human osteoblasts and osteoclasts in coculture. Acta Biomater. 2015;27(294–304). doi: 10.1016/j.actbio.2015.08.042 26318802

[43] Yoshizawa S, Brown A, Barchowsky A, Sfeir C. Magnesium ion stimulation of bone marrow stromal cells enhances osteogenic activity, simulating the effect of magnesium alloy degradation. Acta Biomater. 2014;10(6):2834–42. doi: 10.1016/j.actbio.2014.02.002 24512978

[44] Cecchinato F, Agha NA, Martinez-Sanchez AH, Luthringer BJC, Feyerabend F, Jimbo R, et al. Influence of magnesium alloy degradation on undifferentiated human cells. PLoS One. 2015;10(11):1–18. doi: 10.1371/journal.pone.0142117 26600388 PMC4658158

[45] Leem Yea-Hyun and Lee Kang-Sik and Kim Jung-Hwa and Seok Hyun-Kwang and Chang Jae-Suk and Lee D-H. Magnesium ions facilitate integrin alpha 2-and alpha 3-mediated proliferation and enhance alkaline phosphatase expression and activity in hBMSCs. J Tissue Eng Regen Med. 2016;10:527–36. 24616281 10.1002/term.1861

[46] Maradze D, Musson D, Zheng Y, Cornish J, Lewis M, Liu Y. High Magnesium Corrosion Rate has an Effect on Osteoclast and Mesenchymal Stem Cell Role during Bone Remodelling. Sci Rep [Internet]. 2018;8(1):1–15. Available from: doi: 10.1038/s41598-018-28476-w 29968794 PMC6030161

[47] Zhang X, Zu H, Zhao D, Yang K, Tian S, Yu X, et al. Ion channel functional protein kinase TRPM7 regulates Mg ions to promote the osteoinduction of human osteoblast via PI3K pathway: In vitro simulation of the bone-repairing effect of Mg-based alloy implant. Acta Biomater [Internet]. 2017;63(6):369–82. Available from: doi: 10.1016/j.actbio.2017.08.051 28882757

[48] Burmester A, Willumeit-Römer R, Feyerabend F. Behavior of bone cells in contact with magnesium implant material. J Biomed Mater Res—Part B Appl Biomater. 2015;105(1):165–79. doi: 10.1002/jbm.b.33542 26448207

[49] Wang J, Witte F, Xi T, Zheng Y, Yang K, Yang Y, et al. Recommendation for modifying current cytotoxicity testing standards for biodegradable magnesium-based materials. Acta Biomater. 2015;21:237–49. doi: 10.1016/j.actbio.2015.04.011 25890098

[50] Saldaña L, Bensiamar F, Vallés G, Mancebo FJ, García-Rey E, Vilaboa N. Immunoregulatory potential of mesenchymal stem cells following activation by macrophage-derived soluble factors. Stem Cell Res Ther. 2019;10(1):1–15. 30760316 10.1186/s13287-019-1156-6 PMC6375172

[51] Karnes JM, Daffner SD, Watkins CM. Multiple roles of tumor necrosis factor-alpha in fracture healing. Bone. 2015;78:87–93. doi: 10.1016/j.bone.2015.05.001 25959413

[52] Glass GE, Chan JK, Freidin A, Feldmann M, Horwood NJ, Nanchahal J. TNF-$α$ promotes fracture repair by augmenting the recruitment and differentiation of muscle-derived stromal cells. Proc Natl Acad Sci. 2011;108(4):1585–90. 21209334 10.1073/pnas.1018501108 PMC3029750

[53] Koch AE, Polverini PJ, Kunkel SL, Harlow LA, DiPietro LA, Elner VM, et al. Interleukin-8 as a macrophage-derived mediator of angiogenesis. Science (80-). 1992;258(5089):1798–801. doi: 10.1126/science.1281554 1281554

[54] Yang A, Lu Y, Xing J, Li Z, Yin X, Dou C, et al. IL-8 enhances therapeutic effects of BMSCs on bone regeneration via CXCR2-mediated PI3k/Akt signaling pathway. Cell Physiol Biochem. 2018;48(1):361–70. doi: 10.1159/000491742 30016780

[55] Yoon DS, Lee K-M, Kim S-H, Kim SH, Jung Y, Kim SH, et al. Synergistic action of IL-8 and bone marrow concentrate on cartilage regeneration through upregulation of chondrogenic transcription factors. Tissue Eng Part A. 2016;22(3–4):363–74. doi: 10.1089/ten.tea.2015.0425 26871861

[56] Mondagas SC, Jilka RL. Bone marrow cytokines, and bone remodeling. N Engl J Med. 1995;332:305–11. 7816067 10.1056/NEJM199502023320506

[57] Hameed IA. Using Gaussian membership functions for improving the reliability and robustness of students’ evaluation systems. Expert Syst Appl [Internet]. 2011;38(6):7135–42. Available from: doi: 10.1016/j.eswa.2010.12.048

[58] Sadollah A. Introductory Chapter: Which Membership Function is Appropriate in Fuzzy System? In: Sadollah A, editor. Fuzzy Logic Based in Optimization Methods and Control Systems and Its Applications [Internet]. Rijeka: IntechOpen; 2018.

[59] Iancu I. A Mamdani type fuzzy logic controller. Fuzzy Log Control Concepts, Theor Appl. 2012;325–50.

[60] Romani AM. Cellular magnesium homeostasis. Arch Biochem Biophys [Internet]. 2011;512(1):1–23. Available from: https://www.ncbi.nlm.nih.gov/pmc/articles/PMC3624763/pdf/nihms412728.pdf. 21640700 10.1016/j.abb.2011.05.010 PMC3133480

[61] Mezura-Montes E, Velázquez-Reyes J, Coello Coello CA. A comparative study of differential evolution variants for global optimization. In: Proceedings of the 8th annual conference on Genetic and evolutionary computation. 2006. p. 485–92.

