# Supplementary figures and images for: The osteogenetic activities of mesenchymal stem cells in response to Mg2+ ions and inflammatory cytokines: a numerical approach using fuzzy logic controllers
Source: PLoS Comput Biol. 2022 Sep 15;18(9):e1010482. doi: 10.1371/journal.pcbi.1010482 (PMC9514629; doi:10.1371/journal.pcbi.1010482)

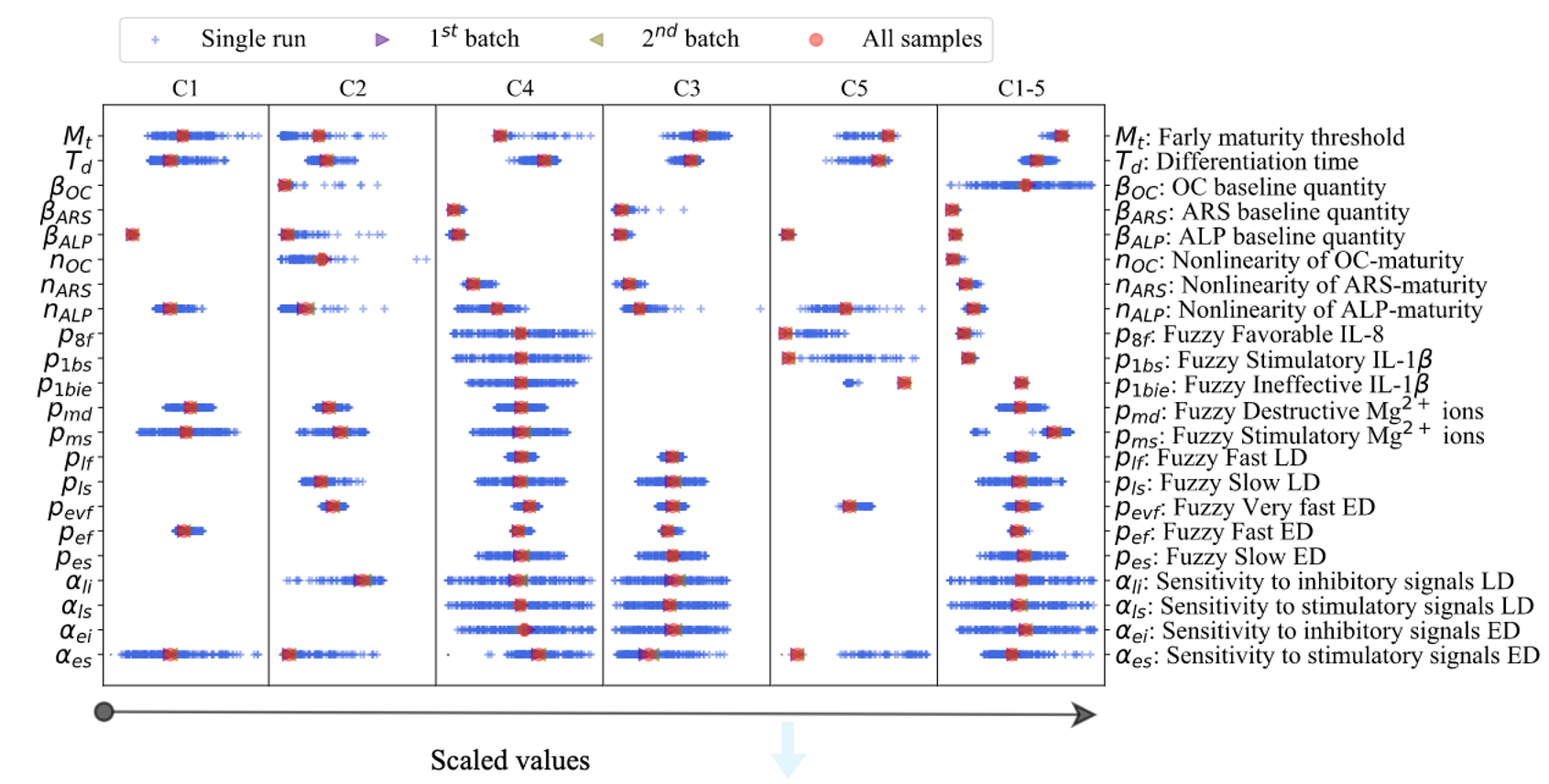

Supplement: S1 Fig — Individual runs are the results of each calibration process; All samples represent the mean of combined individual runs; First (1st) and second (2nd) halfs of samples indicate the means of the frist half and second half of combined individual runs, respectively. The values were scaled by dividing by the length of the priors. (TIFF) [file pcbi.1010482.s001.tiff]
