# Supplementary material for: The osteogenetic activities of mesenchymal stem cells in response to Mg2+ ions and inflammatory cytokines: a numerical approach using fuzzy logic controllers
Source: PLoS Comput Biol. 2022 Sep 15;18(9):e1010482. doi: 10.1371/journal.pcbi.1010482 (PMC9514629; doi:10.1371/journal.pcbi.1010482)
Supplement: S1 Table — (DOCX) [file pcbi.1010482.s002.docx]

| # | Cell type | Experimental variables | Measurements (day) | Measurements | Ref. |
| --- | --- | --- | --- | --- | --- |
| 1 | hBMSCs | **Mg^2+^ ions** (mM)**:** 0.08, 0.8, or 8 | 3,7 | ALP | [1] |
| 2 | HUCPV | **Mg^2+^ ions** (mM): 0.80 or 5.60 | 7, 21 | ALP, OC | [2] |
| 3 | hBMSCs | **IL10** (ng/ml)**:** 0, 0.1, 1, or 10  **TNF-α** (ng/ml)**:** 0, 0.1, 1, or 10 | 14, 21 | ALP, ARS | [3] |
| 4 | hBMSCs | **IL10** (ng/ml): 0, 0.01, 0.1, 1, 10, or 100 | 3,9 | ALP, ARS | [4] |
| 5 | hBMSCs | **IL8** (ng/ml): 0, 1, 10, or 100  **IL1-β** (ng/ml): 0, 1, 10, or 100 | 9 | ALP | [1] |

**References:**

1. Qiao W, Wong KHM, Shen J, Wang W, Wu J, Li J, et al. TRPM7 kinase-mediated immunomodulation in macrophage plays a central role in magnesium ion- induced bone regeneration. Nat Commun [Internet]. 2021;12(2885). Available from: http://dx.doi.org/10.1038/s41467-021-23005-2

2. Luthringer BJC, Willumeit-Römer R. Effects of magnesium degradation products on mesenchymal stem cell fate and osteoblastogenesis. Gene [Internet]. 2016;575(1):9–20. Available from: http://dx.doi.org/10.1016/j.gene.2015.08.028

3. Valles G, Bensiamar F, Maestro-Paramio L, García-Rey E, Vilaboa N, Saldaña L. Influence of inflammatory conditions provided by macrophages on osteogenic ability of mesenchymal stem cells. Stem Cell Res Ther. 2020;11(1):1–15.

4. Chen E, Liu G, Zhou X, Zhang W, Wang C, Hu D, et al. Concentration-dependent, dual roles of IL-10 in the osteogenesis of human BMSCs via P38/MAPK and NF-kB signaling pathways. FASEB J. 2018;32(9):4917–29.
