# Supplementary material for: The osteogenetic activities of mesenchymal stem cells in response to Mg2+ ions and inflammatory cytokines: a numerical approach using fuzzy logic controllers
Source: PLoS Comput Biol. 2022 Sep 15;18(9):e1010482. doi: 10.1371/journal.pcbi.1010482 (PMC9514629; doi:10.1371/journal.pcbi.1010482)
Supplement: S2 Table — The words in green are the cellular inputs while those in red are cellular outputs. (DOCX) [file pcbi.1010482.s003.docx]

| **#** | **Rule** |
| --- | --- |
| 1 | **IF** (TNF-α is Inhibitory) or (IL-10 is Inhibitory) or (Mg^2+^ ions is Inhibitory ED or Destructive) **THEN** Early differentiation is Slow |
| 2 | **IF** (TNF-α is Negligible or Ineffective) and (IL-10 is Negligible) and (IL-8 is Negligible) and (IL-1β is Negligible  or Ineffective) and (Mg^2+^ ions is Physiological or Ineffective) **THEN** Early differentiation is Physiological |
| 3 | **IF** (TNF-α is Stimulatory) or (IL-10 is Favorable) or (Mg^2+^ ions is Stimulatory) **THEN** Early differentiation is Fast |
| 4 | **IF** (IL-10 is Stimulatory) or ((IL-8 is Favorable and IL-1β is Negligible) or (IL-8 is Negligible and IL-1β is Stimulatory) or (IL-8 is Not Negligible and IL-1β is Not Negligible)) **THEN** Early differentiation is Very fast |
| 5 | **IF** (IL-8 is Stimulatory) and (IL-1β is Negligible) **THEN** Early differentiation is Extremely fast |
| 6 | **IF** (TNF-α is Inhibitory) or (IL-10 is Inhibitory) or (Mg^2+^ ions is Inhibitory LD or Destructive) **THEN** Late differentiation is Slow |
| 7 | **IF** (TNF-α is Negligible or Ineffective) and (IL-10 is Negligible) and (Mg^2+^ ions is Not Inhibitory LD) **THEN** Late differentiation is Physiological |
| 8 | **IF** (TNF-α is Stimulatory) or (IL-10 is Favorable) **THEN** Late differentiation is Fast |
| 9 | **IF** (IL-10 is Stimulatory) **THEN** Late differentiation is Very fast |
