# Supplementary material for: The osteogenetic activities of mesenchymal stem cells in response to Mg2+ ions and inflammatory cytokines: a numerical approach using fuzzy logic controllers
Source: PLoS Comput Biol. 2022 Sep 15;18(9):e1010482. doi: 10.1371/journal.pcbi.1010482 (PMC9514629; doi:10.1371/journal.pcbi.1010482)
Supplement: S3 Table — Those marked by ‘-‘were not inferred during that particular calibrations scenario. (DOCX) [file pcbi.1010482.s004.docx]

| **Parameter** | **Label** | **Value range/ prior** | **Reference** | **Inferred value** | | | | | |
| --- | --- | --- | --- | --- | --- | --- | --- | --- | --- |
|  |  |  |  | C1 | C2 | C3 | C4 | C5 | C1-5 |
| $T_{d}$ | Differentiation time | 15-45 days | [1] | 23.1 | 26.5 | 40.2 | 39.6 | 39.9 | 36.2 |
| $M_{t}$ | Early maturity threshold | 0-1 | Estimated | 0.47 | 0.37 | 0.75 | 0.36 | 0.73 | 0.77 |
| $p_{es}$ | Fuzzy *Slow* early differentiation | 0.1-0.4 | Estimated | - | - | 0.25 | 0.25 | - | 0.26 |
| $p_{ef}$ | Fuzzy *Fast* early differentiation | 0.5-0.75 | Estimated | 0.59 | - | 0.57 | 0.60 | - | 0.58 |
| $p_{evf}$ | Fuzzy *Very fast* early differentiation | 0.6-1 | Estimated | - | 0.80 | 0.82 | 0.89 | 0.74 | 0.81 |
| $p_{ls}$ | Fuzzy *Slow* late differentiation | 0.1-0.4 | Estimated | - | 0.19 | 0.26 | 0.25 | - | 0.24 |
| $p_{lf}$ | Fuzzy *Fast* late differentiation | 0.6-0.9 | Estimated | - | - | 0.75 | 0.75 | - | 0.75 |
| $p_{ms}$ | Fuzzy *Stimulatory* Mg^2+^ ions | 2-10 mM | [2] | 5.8 | 6.9 | - | 6.1 | - | 8.1 |
| $p_{md}$ | Fuzzy *Inhibitory* Mg^2+^ ions | 20-40 mM | [3] | 30.9 | 27.8 | - | 30.1 | - | 29.3 |
| $p_{1bie}$ | Fuzzy *Ineffective* IL-1β | 30-199 ng/ml | Estimated | - | - | - | 114.6 | 192.6 | 113.9 |
| $p_{1bs}$ | Fuzzy *Stimulatory* IL-1β | 1-29 ng/ml | Estimated | - | - | - | 15.0 | 1.4 | 3.8 |
| $p_{8f}$ | Fuzzy *Favorable* IL-8 | 1-99 ng/ml | Estimated | - | - | - | 49.8 | 2.2 | 9.9 |
| $\alpha_{es}$ | Sensitivity of the early differentiation rate to the stimulatory signals | 0-20 | Estimated | 7.7 | 1.6 | 6.6 | 12.4 | 2.1 | 8.6 |
| $\alpha_{ei}$ | Sensitivity of the early differentiation rate to the inhibitory signals | 0-20 | Estimated | - | - | 10.6 | 10.5 | - | 10.5 |
| $\alpha_{ls}$ | Sensitivity of the late differentiation rate to the stimulatory signals | 0-20 | Estimated | - | - | 9.6 | 9.9 | - | 9.6 |
| $\alpha_{li}$ | Sensitivity of the late differentiation rate to the inhibitory signals | 0-20 | Estimated | - | 15.8 | 11.2 | 9.6 | - | 9.9 |
| ${}_{ALP}$ | ALP baseline quantity | 0-10 | Estimated | 1.4 | 0.7 | 0.5 | 0.7 | 0.4 | 0.4 |
| ${}_{ARS}$ | ARS baseline quantity | 0-10 | Estimated | - | - | 0.6 | 0.4 | - | 0.2 |
| ${}_{OC}$ | OC baseline quantity | 0-10 | Estimated | - | 0.4 | - | - | - | 5.3 |
| $n_{ALP}$ | Degree of nonlinearity between ALP and maturity | 0-10 | Estimated | 3.8 | 2.4 | 2.1 | 3.4 | 4.4 | 1.7 |
| $n_{ARS}$ | Degree of nonlinearity between ARS and maturity | 0-10 | Estimated | - | - | 1.3 | 1.7 | - | 1.1 |
| $n_{OC}$ | Degree of nonlinearity between OC and maturity | 0-10 | Estimated | - | 4.0 | - | - | - | 0.2 |
| $k_{\begin{aligned} ALP, \\ 1,5 \end{aligned}}$ | Correction coefficient applied to map maturity to ALP for study 1 | 0-200 | Estimated | - | - | - | - | 93.7 | 31.7 |
| $k_{ALP,2}$ | Correction coefficient applied to map maturity to ALP for study 2 | 0-1 | Estimated | - | 0.37 | - | - | - | 0.64 |
| $k_{OC,2}$ | Correction coefficient applied to map maturity to OC for study 2 | 0-1 | Estimated | - | 0.49 | - | - | - | 0.39 |
| $k_{ALP,3}$ | Correction coefficient applied to map maturity to ALP for study 2 | 0-10 | Estimated | - | - | - | 5.1 | - | 6.9 |
| $k_{ARS,3}$ | Correction coefficient applied to map maturity to ARS for study 2 | 0-10 | Estimated | - | - | - | 2.3 | - | 3.3 |
| $k_{ALP,4}$ | Correction coefficient applied to map maturity to ALP for study 3 | 0-1000 | Estimated | 491.2 | - | 304.3 | - | - | 295.1 |
| $k_{ARS,4}$ | Correction coefficient applied to map maturity to ARS for study 3 | 0-1000 | Estimated | - | - | 482.1 | - | - | 691.1 |

**References:**

1 . Baksh D, Yao R, Tuan RS. Comparison of proliferative and multilineage differentiation potential of human mesenchymal stem cells derived from umbilical cord and bone marrow. Stem Cells. 2007;25(6):1384–92.

2. Zhang X, Zu H, Zhao D, Yang K, Tian S, Yu X, et al. Ion channel functional protein kinase TRPM7 regulates Mg ions to promote the osteoinduction of human osteoblast via PI3K pathway: In vitro simulation of the bone-repairing effect of Mg-based alloy implant. Acta Biomater [Internet]. 2017;63(6):369–82. Available from: https://doi.org/10.1016/j.actbio.2017.08.051

3. Burmester A, Willumeit-Römer R, Feyerabend F. Behavior of bone cells in contact with magnesium implant material. J Biomed Mater Res - Part B Appl Biomater. 2015;105(1):165–79.
