# Supplementary material for: The osteogenetic activities of mesenchymal stem cells in response to Mg2+ ions and inflammatory cytokines: a numerical approach using fuzzy logic controllers
Source: PLoS Comput Biol. 2022 Sep 15;18(9):e1010482. doi: 10.1371/journal.pcbi.1010482 (PMC9514629; doi:10.1371/journal.pcbi.1010482)
Supplement: S1 Text — (DOCX) [file pcbi.1010482.s005.docx]

Scikit Fuzzy was employed for the development of the fuzzy-logic controller [1]. barneySA, a free Python package developed in-house, was used for large-scale simultaneous perturbation (LSSP) analysis [2]. Custom Python script was developed to conduct small-scale simultaneous perturbation. The differential evolution package of Scipy in Python was used for the parameter estimation. We used Message Passing Interface (MPI) to manage large computational demands. Maxwell computational resources operated at Deutsches Elektronen-Synchrotron (DESY) are used with more than 100 CPUs for the computations.

**References**

1. Warner J, Sexauer J, scikit-fuzzy, twmeggs, alexsavio, Unnikrishnan A, et al. JDWarner/scikit-fuzzy: Scikit-Fuzzy version 0.4.2 [Internet]. Zenodo; 2019. Available from: https://doi.org/10.5281/zenodo.3541386

2. Nourisa J. janursa/barneySA v1.0.1. 2021 Feb 19 [cited 2021 Feb 19]; Available from: https://doi.org/10.5281/zenodo.4552580#.YDAI3hxOa0k.mendeley
